# ZNF503-AS2 is a promising therapeutic target and is associated with the immune microenvironment in glioma

**DOI:** 10.1371/journal.pone.0314618

**Published:** 2024-12-02

**Authors:** Yibo Wu, Guangjing Mu, Fang Li, Yanfei Sun, Xiaoying Lin, Xuemeng Liu, Zhimin Zhao, Mingzhi Han, Donghai Wang, Bin Huang, Xingang Li

**Affiliations:** 1 Department of Neurosurgery, Jinan Microecological Biomedicine Shandong Laboratory and Shandong Key Laboratory of Brain Function Remodeling, Cheeloo College of Medicine and Institute of Brain and Brain-Inspired Science, Qilu Hospital, Shandong University, Jinan, China; 2 Shandong Key Laboratory of Brain Function Remodelling, Jinan, China; 3 Department of Health Care, Jinan Central Hospital, Jinan, China; 4 The Second Hospital, Cheeloo College of Medicine, Shandong University, Jinan, China; 5 Medical Integration and Practice Center, Cheeloo College of Medicine, Shandong University, Jinan, China; 6 Department of Neurosurgery, Qilu Hospital of Shandong University Dezhou Hospital, Dezhou, China; Tehran University of Medical Sciences, ISLAMIC REPUBLIC OF IRAN

## Abstract

**Background:**

Glioma is the most common intracranial malignancy, and the available treatment options are poor. Long noncoding RNAs (lncRNAs) have been reported to be involved in the malignant progression of glioma. The role of ZNF503-AS2 in glioma has not been reported.

**Methods:**

We screened ZNF503-AS2 with upregulated expression in glioblastoma (GBM) by analyzing the TCGA, CGGA and GTEx databases. Single sample gene set enrichment analysis (ssGSEA) was used to calculate the enrichment of immune cells and signaling pathways in glioma samples. Single-cell datasets were used to analyze the distribution of ZNF503-AS2. In vitro experiments were used to investigate the biological function of ZNF503-AS2.

**Results:**

ZNF503-AS2 was highly expressed in glioma and was associated with poor prognosis, malignant progression and infiltration of immunosuppressive cells. Single-cell transcriptomic analysis showed that ZNF503-AS2 was mainly expressed in macrophages and tumor cells. Further analysis revealed that immunotherapy may have better efficacy in patients with low ZNF503-AS2 expression. In vitro experiments showed that knockdown of ZNF503-AS2 reduced the proliferation, invasion and migration ability of glioma cells, induced G2/M cell cycle arrest and promoted apoptosis.

**Conclusions:**

ZNF503-AS2 might be a valuable biomarker for predicting the prognosis of glioma patients and a potential target for glioma therapy.

## Introduction

Gliomas are the most common intracranial malignant tumors [1], with a high recurrence rate and high drug resistance, and complete surgical removal of glioma is difficult [2,3]. World Health Organization (WHO) grade II and WHO grade III gliomas are defined as lower-grade glioma (LGG). WHO grade IV glioma is defined as GBM [4], and the existing treatment methods are not effective in improving the prognosis of patients with GBMs, with a survival time of approximately 14 months [5]. Recurrent glioma progress more rapidly and patients with glioma of the mesenchymal (MES) subtype have a worse prognosis [6–8].

Immunotherapy is currently a promising therapeutic approach for intercranial malignant tumors and has been demonstrated to have significant efficacy in the treatment of other tumors, but its efficacy in glioma is still unsatisfactory [2,9–11]. The complex immune microenvironment of glioma is the main factor affecting immunotherapy efficacy [12,13]. The immunosuppressive microenvironment contributes to the immune tolerance of tumor cells and eventually immune escape, which greatly increases the difficulty of treatment, so research on the microenvironment of glioma is particularly important for the treatment of glioma [14–16]. Due to the drug resistance of glioma, immunotherapy has shown good efficacy in only a few patients. With the wide application of bioinformatics, important biomarkers such as isocitrate dehydrogenase 1 (IDH1) mutations and 1p/19q deletions have been discovered. However, more important biomarkers are still to be discovered, and the poor efficacy of immunotherapy is also related to the lack of specific biomarkers. Therefore, the search for valuable biomarkers for glioma treatment remains necessary [17,18].

Initially, non-coding RNAs were labeled as nonfunctional RNA fragments because they could not be translated into proteins. However, with an increasing number of studies, lncRNAs have been found to be able to influence gene expression and transcription and can promote tumor growth and recurrence by affecting cell proliferation and invasion [19–21]. In addition, many studies have reported that lncRNAs can affect the tumor microenvironment (TME) by participating in inflammatory, stromal, immune, and other related pathways, but the role of lncRNAs in glioma has not been extensively studied [22,23].

ZNF503-AS2 is localized on chromosome 10, and previous studies have only reported the effect of ZNF503-AS2 in kidney renal clear cell carcinoma [24]; its function in glioma remains unknown.

In this study, we used datasets from TCGA and CGGA to study the role of ZNF503-AS2 in glioma and its relationship with prognosis, molecular subtype, immune microenvironment, inflammatory response, and immune checkpoint blockade (ICB) treatment. The role of ZNF503-AS2 in glioma cells was verified by in vitro functional experiments. In conclusion, these results showed that ZNF503-AS2 may serve as a reliable biomarker for predicting the prognosis of glioma patients and distinguishing molecular subtypes. Targeting ZNF503-AS2 in a therapeutic approach seems to benefit glioma patients and may serve as a potential target for immunotherapy.

## Materials and methods

### Data sources

The mRNA sequencing data and clinical characteristics were downloaded from the TCGA database (http://cancergenome.nih.gov/) and labeled for the training cohort. mRNA sequencing data and clinical information from two cohorts were downloaded from the CGGA database (http://www.cgga.org.cn/) for validation, labeled CGGA325 and CGGA693 in the article. Sequencing data for normal brain tissues were downloaded from the GTEx database (https://xenabrowser.net/datapages/). In addition, the differential expression of ZNF503-AS2 in normal brain tissues and glioma was validated using the GSE131273 database (https://www.ncbi.nlm.nih.gov/). ICB data for GBM were obtained from GSE121810, ICB data for skin cutaneous melanoma (SKCM) were obtained from GSE78220 and GSE91061, and ICB data for non-small cell lung cancer (NSCLC) were obtained from GSE135222. Based on the median value of ZNF503-AS2 expression, we divided the samples into high and low-expression groups.

### Analysis of the TME

We used ssGSEA to analyze the score of immune cells and immune function and classified the samples into Cluster 1 and Cluster 2 based on the results of ssGSEA.

### Enrichment analysis of signaling pathways

To study signaling pathway activation in different samples, we downloaded gene sets from the Molecular Signatures Database (MSigDB) and used the "GSVA" R package to calculate enrichment scores and draw heatmaps. We also imputed differentially expressed genes (DEGs) between the high and low-expression groups into Metascape (http://Metascape.org/gp/index.html#/) for transcription factor prediction.

### Single-cell data analysis

TISCH2 (http://tisch.comp-genomics.org) collected tumor single-cell transcriptomic data and clinical information of patients and processed the data in a unified way, and we downloaded single-cell data through TISCH2 for analysis.

### Methylation analysis

We downloaded methylation data from cBioPortal (https://www.cbioportal.org/) and analyzed the relationship between ZNF503-AS2 methylation and ZNF503-AS2 expression using Pearson correlation.

### Sangerbox tools

We used Sangerbox tools (http://www.sangerbox.com/tool) to validate the expression of ZNF503-AS2 in pancancer and to correlate ZNF503-AS2 with RNA modification genes, cancer stemness index, loss of heterozygosity (LOH), mutant-allele tumor heterogeneity (MATH), tumor ploidy, homologous recombination deficiency (HRD) and prognosis.

### Construction of a ceRNA network

The lncRNA‒miRNA and miRNA‒mRNA target data were obtained from miRcode (http://www.mircode.org/index.php), and based on the expression differences between tumor tissue samples and normal tissue samples, a total of 5 miRNAs and 40 mRNAs were screened together with ZNF503-AS2 to construct the ceRNA network.

### Drug sensitivity prediction

We used the GDSC dataset (https://www.cancerrxgene.org/) and the pRRophetic algorithm to predict drug sensitivity in glioma samples. The 30 sensitive and insensitive drugs with the strongest correlations and the pathways targeted by these drugs were mapped.

### Immunotherapy prediction

We used the TIDE tool (http://tide.dfci.harvard.edu/) to calculate a TIDE score for each sample to predict response to ICB [25].

### Cell lines

A172, LN229, and U118 cell lines were purchased from ATCC and cultured in Dulbecco’s modified Eagle medium (Thermo Fisher Scientific; Waltham, MA, USA) with 10% fetal bovine serum (FBS; Thermo Fisher Scientific) and 1% penicillin-streptomycin mixture, and patient-derived GBM stem-like cells (GSCs) P3 were isolated and identified from GBM surgical specimens and cultured in Neurobasal’s medium (Gibco/Thermo Fisher Scientific) with 10 ng/ml basic fibroblast growth factor (bFGF; PeproTech), 20 ng/ml epidermal growth factor (EGF; PeproTech; East Windsor, NJ, USA) and 2% B-27 mixed culture (Thermo Fisher Scientific). All cells were cultured at 37°C in an incubator with 5% CO_2_, and the culture medium was changed periodically.

### Cell transfection

SiRNA and Lipofectamine 3000 reagent were used for transient transfection of LN229, U118, A172, and GBM#P3 cells and were divided into negative control (NC), si-1, and si-2 groups. siRNAs targeting ZNF503-AS2 were sequenced as follows: si-ZNF503-AS2-1: 5’-GCUGCAAGUUAGCAAGAGATT-3’; si-ZNF503-AS2-2: 5’-CUUCGAGCCAGUGCUUAAATT-3’; si-NC: 5’-UUCUCCGAACGUGUCACGUTT-3’.

### qRT‒PCR

Cells were transfected for 48 h, and total RNA was extracted from the cells using the RNA-Quick Purification Kit (#RN001, ESscience Biotech). Extracted RNA was reverse transcribed to cDNA using Hifair^®^ III 1st Strand cDNA Synthesis SuperMix (Yeasen Biotechnology, #11141ES10), Hieff^®^ qPCR SYBR Green Master Mix (Yeasen Biotechnology, #11201ES03) was then applied for qRT‒PCR, and GAPDH was used as a control to normalize the data. The primers targeting ZNF503-AS2 were F, CATAAATCGCGGGTGTCAGG; R, ATTTGGTGTCTCGTCCCCG; primers for GAPDH were F, GCACCGTCAAGGCTGAGAAC; R, TGGTGAAGACGCCAGTGGA.

### CCK-8 assay

Cells were uniformly seeded in 96-well plates (3000 cells per well), transfected after 24 h, and divided into three groups (NC, si-1, si-2) for the CCK-8 assay according to the manufacturer’s protocols (Yeasen Biotechnology, #40203ES76) at 24 h, 48 h, 72 h, and 96 h, respectively.

### Colony forming assay

The treated cells were uniformly seeded in 6-well plates (600 cells per well) and cultured in an incubator at 37°C and 5% CO2 for 10–14 days. When the cells had grown to a suitable density, they were placed in 4% paraformaldehyde for 15 min for fixation and stained with crystal violet for 15 min for counting and imaging.

### Cell invasion and migration assay

For transwell assays, after the treated cells were grouped, the cells were added to the upper chamber (2000/well) together with DMEM without FBS, and the lower chamber was filled with medium containing 10% FBS. After incubation for 24–48 hours, the cells in the upper chamber were wiped off with a cotton swab, and the cells were fixed in 4% paraformaldehyde for 15 minutes and stained with crystal violet for 15 minutes. The cells that had penetrated the membranes were visualized and imaged under a microscope. For wound healing assays, the transfected LN229 and A172 cells were inoculated in 6-well plates. The monolayers were scratched with a pipette tip and incubated in DMEM without serum for 48 h. Migrated distances were analyzed using ImageJ software.

### EdU assay

The EdU assay was performed according to the manufacturer’s protocol (Yeasen Biotechnology, #40275ES60). Images were obtained using a Leica inverted fluorescence microscope.

### Flow cytometry

For cell cycle analysis, treated cells were dehydrated and fixed in 70% ethanol for 48 h, centrifuged to remove the 70% ethanol, stained with PI (BD Biosciences; Franklin Lakes, NJ, USA) for 15 min, and then flow cytometry was used to detect the cell cycle. For apoptosis, after cell collection, cells were washed with precooled PBS and resuspended by adding 100 μL of 1× binding buffer. Then, 5 μL of Annexin V-FITC and 10 μL of PI (Yeasen Biotechnology, #40302ES50) were added and mixed well, and the reaction was avoided from light and at room temperature for 10–15 min before adding 400 μL of 1× binding buffer. Apoptosis was detected by flow cytometry.

### Statistical analysis

Statistical analyses were completed using R (version 4.2.0) and GraphPad Prism (version 8.0). The Mann-Whitney test was used for comparison of data that did not obey a normal distribution. Student’s t test was used for normally distributed data. ANOVA was used for multiple comparisons, survival analysis was performed using the Kaplan–Meier method, and the log-rank test was used to compare survival differences between the two groups. The "maftools" package was used to visualize the tumor mutation burden (TMB) in the high and low-expression groups, and correlation analysis was performed using the Spearman test. Three independent experiments were performed. Data for each group were represented as the mean standard error of the mean (SEM). All tests were two-sided, with *P* > 0.05 considered not statistically significant (ns) and *P <* 0.05 considered statistically significant.

### Ethics statement

All the protocols in our study were admitted by the Research Ethics Committee of Shandong University and the Ethics Committee of Qilu Hospital (Shandong, China) (SDULCLL2021-2-26). All experiments and analyses were performed under the guidance of corresponding protocols or guidelines.

## Results

### ZNF503-AS2 expression is upregulated in glioma and correlated with molecular subtype

**Fig 1:**
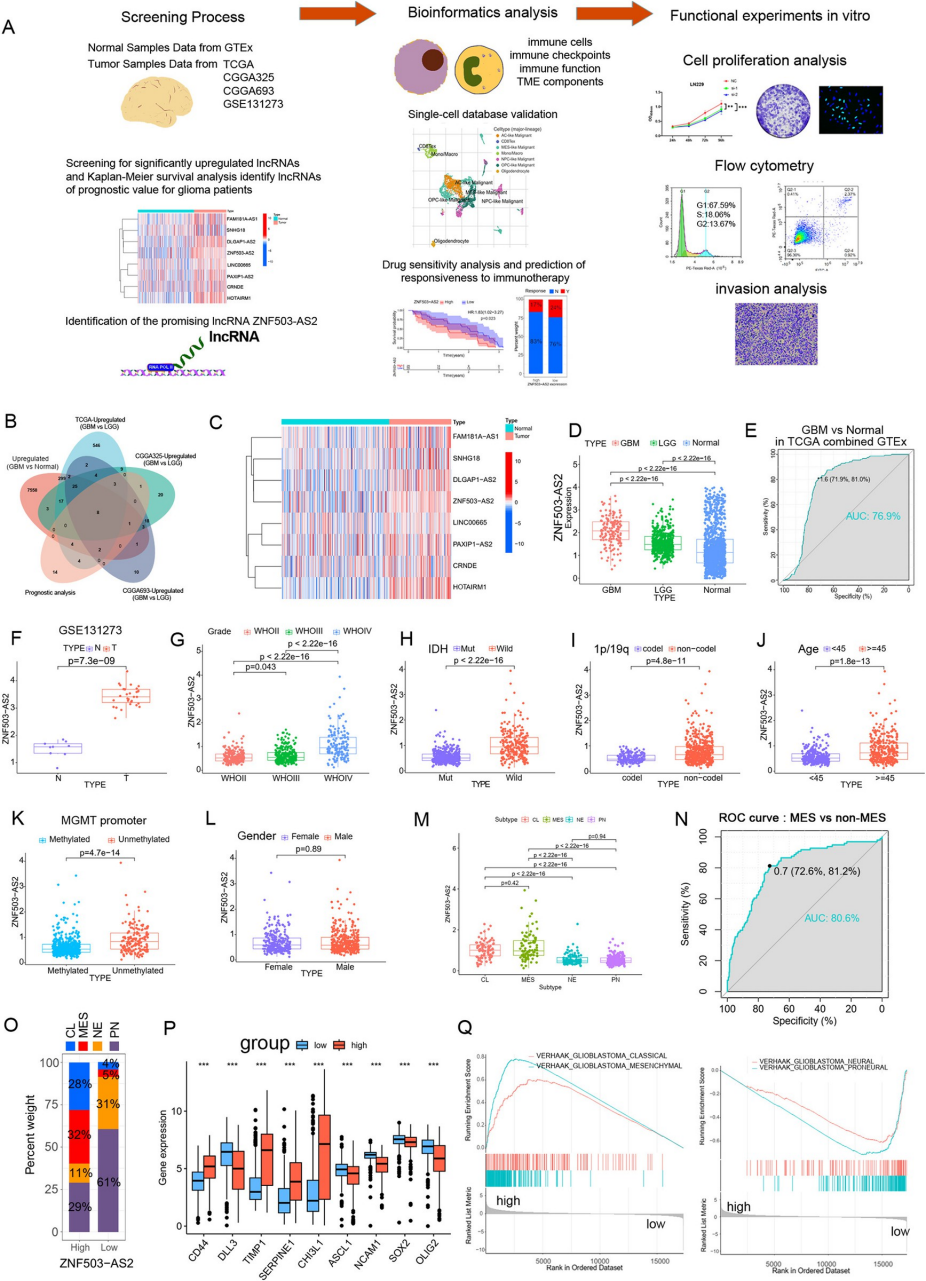
Association of ZNF503-AS2 expression with different clinical features. (A) Article research process. (B) Screen ZNF503-AS2 from the TCGA, CGGA, and GTEx databases. (C) The heatmap shows valuable lncRNAs. (D) ZNF503-AS2 expression is upregulated in GBM and LGG. (E) ROC curve analysis of ZNF503-AS2 predicts GBM in TCGA combined with the GTEx database. (F) ZNF503-AS2 expression is upregulated in glioma in the GSE131273 database. (G-M) Distribution of ZNF503-AS2 in different WHO grades (G), IDH statuses (H), 1p/19q statuses (I), ages (J), MGMT promoter statuses (K), sexes (L), and molecular subtypes (M). (N) The ROC curves indicated that ZNF503-AS2 had high accuracy in the prediction of the MES subtype. (O) Distribution of different molecular subtypes between the two groups. (P) Differential expression of MES and PN markers between the two groups. (Q) GSEA showed that samples with high expression of ZNF503-AS2 were enriched in the MES subtype and CL subtype, and samples with low expression of ZNF503-AS2 were enriched in the NE subtype and PN subtype. NS, not statistically significant; * *P* < 0.05; ** *P* < 0.01; *** *P* < 0.001.

The research content and process of this paper are shown in Fig 1A. In order to find valuable lncRNAs, we first used the TCGA database to screen out lncRNAs with upregulated expression in GBM compared with normal brain tissues and then performed differential analysis on GBM (n = 169) and LGG (n = 535) samples to further screen the lncRNAs that were upregulated in GBM in the TCGA database. Then the same method was used to differentially analyze the GBM and LGG samples in the CGGA325 database (including 139 GBM samples and 182 LGG samples) and the CGGA693 database (including 229 GBM samples and 443 LGG samples). The intersection of the results was taken to obtain 33 valuable lncRNAs, and the final 8 lncRNAs were obtained by the prognostic analysis (Fig 1B). We summarized these 8 lncRNAs with heatmaps in the TCGA combined GTEx database (Fig 1C), whereas the roles of CRNDE, SNHG18, etc. in glioma had already been reported, while the role of ZNF503-AS2 in glioma remained unknown. Therefore, we only studied ZNF503-AS2 in this paper.

By comparing normal brain tissue with GBM in TCGA, ZNF503-AS2 expression was elevated in GBM (S1A Fig) and the area under the curve (AUC) value of the receiver operating characteristic (ROC) curve was 0.822 (S1B Fig). We further combined data from normal brain tissue in GTEx to validate this conclusion and the AUC value was 0.769 (Fig 1D and 1E). And we found that this difference was significant in LGG as well as in all glioma (S1C–S1E Fig). The results of the analysis of the GSE131273 database validated our conclusions (Fig 1F). ZNF503-AS2 expression correlated with patient WHO grade, IDH mutation, 1p/19q codeletion, age, and MGMT promoter status but not sex (Fig 1G–1L).

Molecular subtypes are helpful in predicting malignancy in glioma, and patients with the MES subtype and classical (CL) subtype usually have a worse prognosis than those with the proneural (PN) and neural (NE) subtypes [26,27]. The analysis showed that ZNF503-AS2 was expressed at a higher level in the MES and CL subtypes (Fig 1M) and was positively correlated with the enrichment scores of the MES and CL subtypes and negatively correlated with the enrichment scores of the NE and PN subtypes (S1F Fig). The results of ROC curve analysis showed that the expression of ZNF503-AS2 could better distinguish patients with the MES subtype from those with the non-MES subtype (AUC = 0.806; Fig 1N), and the proportion of patients with the MES subtype was much higher in the high-expression group (32% vs. 5%; Fig 1O). We further analyzed the expression of MES and PN markers and found that MES markers were elevated in the high-expression group and PN markers in the low-expression group (Fig 1P). The key markers CD44 (R = 0.44, *P* < 0.001) and CHI3L1 (R = 0.5, *P* < 0.001) also showed correlation with ZNF503-AS2 (S1G and S1H Fig) and a similar trend was observed for MES signature genes (S1I Fig). GSEA showed that the samples with high expression of ZNF503-AS2 were enriched in the MES and CL subtypes and that the low-expression samples were enriched in the NE and PN subtypes (Fig 1Q). We validated our findings in the CGGA325 and CGGA693 databases that ZNF503-AS2 showed a correlation with clinical characteristics and an association with MES molecular subtypes (S2 Fig), and the ROC curves indicated a good predictive ability for GBM, IDH mutations, 1p/19q codeletion, and molecular subtypes with poor prognosis (S3 Fig).

### High expression of ZNF503-AS2 predicted poor prognosis

**Fig 2:**
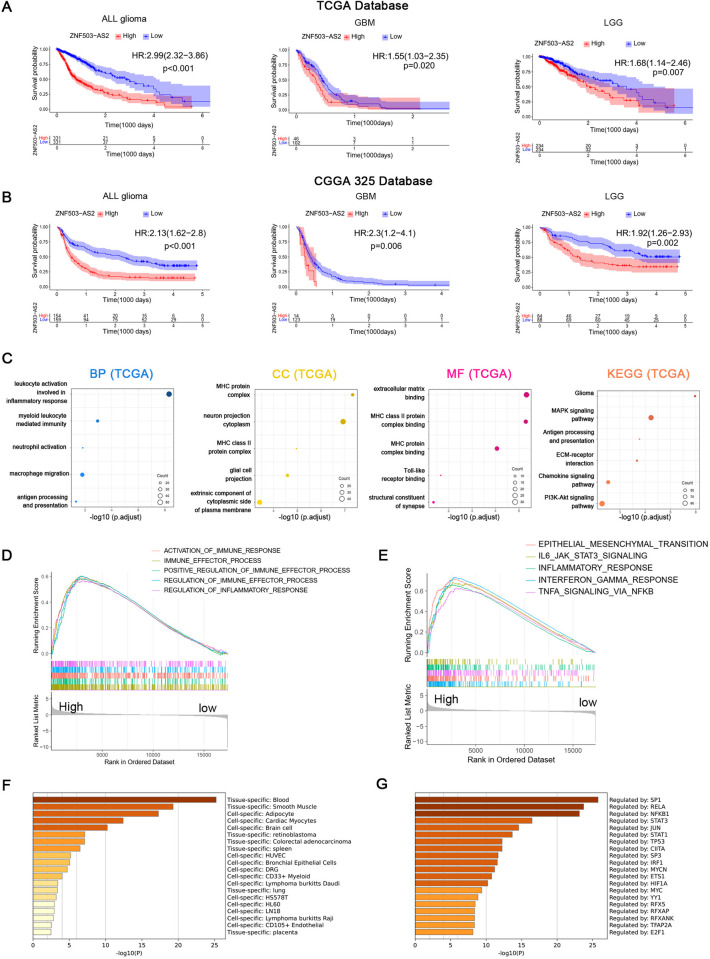
Effect of ZNF503-AS2 on prognosis and function in glioma. (A, B) Survival analysis based on ZNF503-AS2 expression in the TCGA (A) and CGGA325 databases (B). (C) Enrichment analysis of DEGs in the TCGA database. (D, E) GSEA of DEGs in GO biological processes (D) and hallmark gene sets (E) in the TCGA database. (F) PaGenBase shows that the DEGs are highly specifically expressed in the blood and spleen in the TCGA database. (G) TRRUST shows that DEGs are mainly regulated by SP1, RELA and NFKB1 in the TCGA database.

To explore the role of ZNF503-AS2 in glioma prognosis, we analyzed CGGA325 glioma datasets, and the results of univariate Cox regression analyses showed that tumor type, WHO grade, age, and ZNF503-AS2 expression were risk factors, whereas IDH mutation and 1p/19q co-deletions were protective factors. The results of multivariate Cox regression analysis verified that tumor type, WHO grade, age, and ZNF503-AS2 expression were independent risk factors affecting the prognosis of glioma (S4A Fig). The same analysis is available in the CGGA693 database (S4B Fig). Kaplan–Meier survival analysis of both the TCGA and CGGA325 datasets showed that patients with high expression of ZNF503-AS2 in all glioma, GBM, and LGG had a poorer prognosis (Fig 2A and 2B). The ROC curves showed that ZNF503-AS2 had high accuracy in predicting the overall survival (OS) of glioma patients at 1, 3, and 5 years (S4C Fig). Similar trends were obtained by analyzing the CGGA693 database (S4D Fig). In addition, we constructed a nomogram by combining clinical characteristics, patient survival information and the expression of ZNF503-AS2. The C-index of this nomogram model was 0.779 (S4E Fig), and the results of the calibration curve also showed that the predicted survival results were highly consistent with the actual results (S4F Fig), indicating that the prediction map had good prediction accuracy.

### Functional enrichment analysis of DEGs

To investigate the effect of ZNF503-AS2 on glioma function, we divided the glioma samples into high and low-expression groups according to the median value of ZNF503-AS2 expression and identified the DEGs between the two groups (|log2 (fold change)| > 2 and *P* < 0.05). These DEGs were used to perform Gene Ontology (GO) enrichment analysis and Kyoto Encyclopedia of Genes and Genomes (KEGG) enrichment analysis and were ranked according to *p*.*adjust*. GO enrichment results are mainly related to tumor immunity, such as antigen processing and presentation, macrophage migration, leukocyte activation involved in the inflammatory response, major histocompatibility complex (MHC) protein complex, and Toll-like receptor binding. KEGG results showed that these DEGs were significantly enriched in glioma signaling pathway, MAPK signaling pathway, ECM-receptor interaction, and PI3K-Akt signaling pathway (Fig 2C). In addition, we also performed GSEA to explore the potential link between ZNF503-AS2 and glioma, and the results showed that the function of ZNF503-AS2 was associated with tumor immune-related pathways, especially in inflammatory response and regulation of the immune response (Fig 2D). The results of the enrichment analysis of the "Hallmark" gene set confirmed our conclusion that the stromal cell activation pathway, inflammatory response pathway and interferon signaling pathway were upregulated in the high-expression group (Fig 2E). Enrichment analysis of these DEGs in PaGenBase showed that DEGs were highly expressed in tissues with a high distribution of peripheral immune cells, such as blood and spleen, suggesting that ZNF503-AS2 may influence glioma progression by promoting immune cell infiltration into the tumor (Fig 2F). In addition, we found that these genes were mainly regulated by SP1, RELA, and NF-kB1. SP1 played an important role in drug resistance and tumor growth in glioma, and RELA and NF-kB1 are involved in carcinogenic activation through the NF-kB signaling pathway through enrichment analysis of the Transcriptional Regulatory Relationships Unraveled by Sentence-based Text mining (TRRUST) database (Fig 2G). There is a similar trend in the CGGA325 database (S4G–S4K Fig).

### ZNF503-AS2 is involved in remodeling the immune microenvironment of glioma

**Fig 3:**
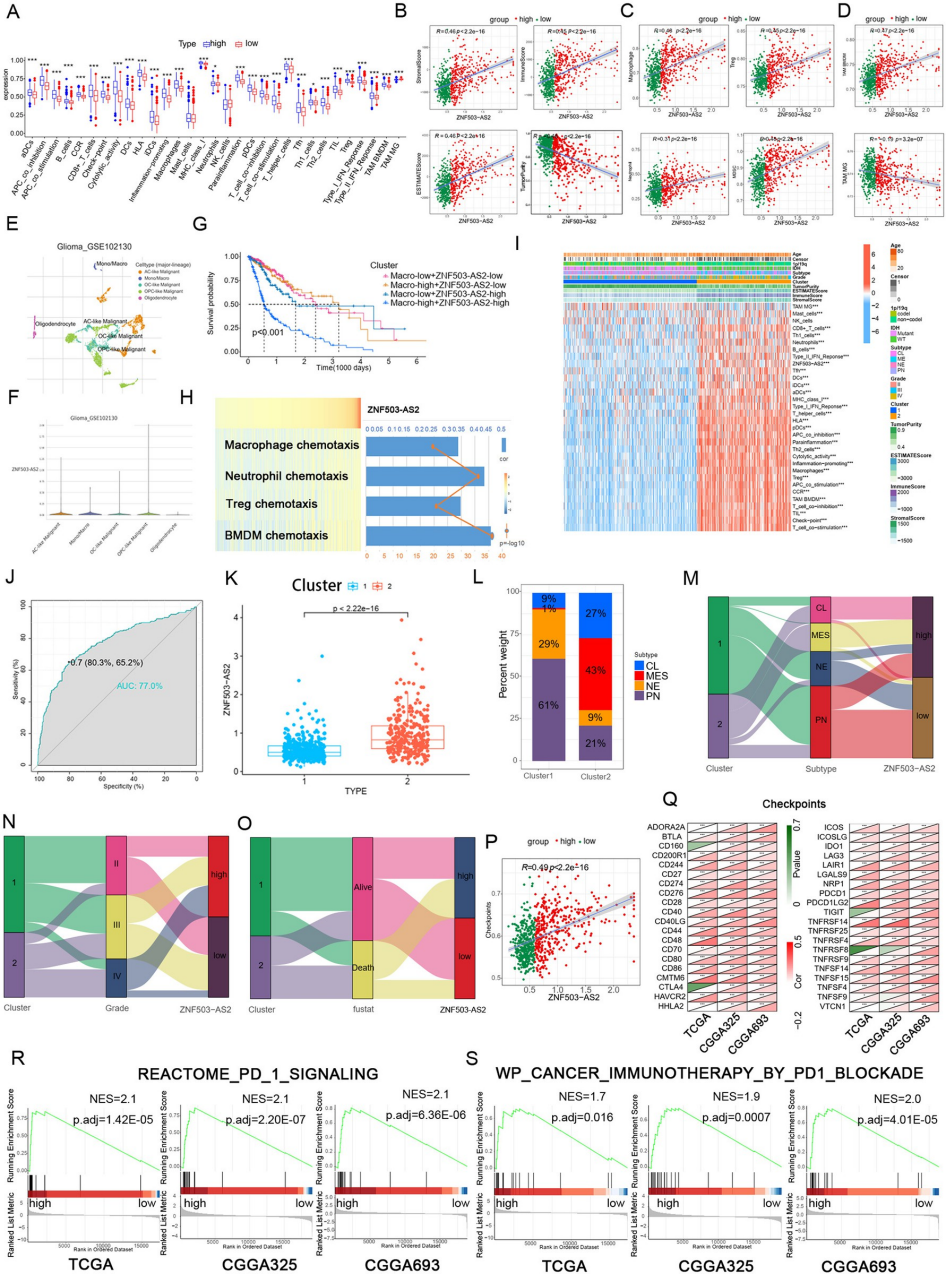
ZNF503-AS2 is associated with the TME in glioma. (A) ssGSEA showed significant differences in immune cell and immune function enrichment scores between the two groups. (B) Correlation analysis of ZNF503-AS2 with stromal, immune, ESTIMATE scores, and tumor purity. (C) Correlation analysis of immunosuppressive cells with ZNF503-AS2. (D) Correlation analysis of two TAMs with ZNF503-AS2. (E, F) Analysis of the GSE102130 single-cell dataset showed that ZNF503-AS2 was predominantly expressed in tumor cells and macrophages. Single-cell data and images were obtained from TISCH2. (G) Survival analysis of combined macrophage infiltration with ZNF503-AS2 expression. (H) Heatmap of the correlation between the chemotaxis of immunosuppressed cells and ZNF503-AS2 expression. (I) Heatmap of immune typing pattern based on ZNF503-AS2, immune cells and immune function. (J) ROC curves showed the high accuracy of ZNF503-AS2 in predicting the two immune typing patterns. (K) The expression of ZNF503-AS2 was different between the two immune typing patterns. (L) Distribution of molecular subtypes in two immune typing patterns. (M-O) Alluvial diagram showing that MES molecular subtypes (M), GBM (N), and dead patients (O) were mostly distributed in the Cluster 2 and ZNF503-AS2 high-expression groups. (P) The enrichment score of immune checkpoints was significantly positively correlated with ZNF503-AS2. (Q) Correlation analysis of immune checkpoint molecules with ZNF503-AS2. (R, S) GSEA showed that samples with high ZNF503-AS2 expression were enriched in the PD1 signaling pathway (R) and the PD1-blocked immunotherapy pathway (S). NS, not statistically significant; * *P* < 0.05; ** *P* < 0.01; *** *P* < 0.001.

To further investigate the role of ZNF503-AS2 in the TME, we analyzed the gene set containing immune cells and immune functions, and the results showed that the high-expression group had higher immune cell infiltration and higher immunosuppression (Fig 3A), as well as higher stromal and immune scores and lower tumor purity (Fig 3B), suggesting that there may be a close link between ZNF503-AS2 and the TME of glioma. According to previous reports, the complex TME is largely responsible for the difficulty of glioma treatment, and the suppressive immune microenvironment seriously affects the effectiveness of immunotherapy. We analyzed the correlation between ZNF503-AS2 and immunosuppressive cells (macrophages, neutrophils, Tregs and MDSCs), and the results showed all positive correlations (Fig 3C). We found that ZNF503-AS2 was mainly associated with promoting the infiltration of bone marrow-derived macrophages (BMDMs) rather than tissue-resident microglias (MGs) (Fig 3D) [28,29]. We found that ZNF503-AS2 was not only highly expressed in tumor tissues but also had high levels in macrophages (Fig 3E and 3F) by analyzing the single cell dataset GSE102130. We further combined ZNF503-AS2 and macrophages for analysis and found that ZNF503-AS2 could predict the prognosis of patients with high accuracy without relying on macrophage infiltration (Fig 3G). Patients with low ZNF503-AS2 expression had a better prognosis when the degree of tumor-associated macrophages (TAMs) infiltration was high, suggesting that ZNF503-AS2 may serve as a valid target for patients with high macrophage infiltration. We also found that ZNF503-AS2 positively correlated with the chemotaxis of immunosuppressive cells (Fig 3H). Using the same method to validate the results in the CGGA325 and CGGA693 databases, the results were consistent with our prediction that ZNF503-AS2 positively correlated with multiple molecular markers of immunosuppressive cells (S5 Fig), promoted immunosuppressive cell infiltration, and helped to shape the immunosuppressive TME (S6 Fig).

To further understand the function of ZNF503-AS2 in tumor immunity, we classified glioma samples based on ZNF503-AS2 expression, immune cells, and immune function gene sets and identified two immune typing patterns, which were labeled Cluster 1 and Cluster 2 (Fig 3I). Tumor immune-related functions were upregulated in Cluster 2, and the ROC curve indicated that ZNF503-AS2 had high accuracy in predicting immune typing patterns, with an AUC of 0.77 (Fig 3J). Further analysis showed elevated ZNF503-AS2 expression in Cluster 2 (Fig 3K) and a significant difference in the distribution of highly malignant MES molecular subtypes between the two immune typing patterns (Fig 3L and 3M), as well as more GBM samples and dead patients in Cluster 2 type (Fig 3N and 3O). According to previous reports, immune checkpoint molecules play an important role in immunotherapy, and high expression of immune checkpoints suppresses the immune function of the body, which ultimately leads to immune escape. We found that ZNF503-AS2 was positively correlated with the enrichment scores of immune checkpoints (Fig 3P), and the correlation analysis results were consistent with the prediction in all three datasets (Fig 3Q). In addition, we found that the DEGs were significantly enriched in the PD1 signaling pathway and immunotherapy by the PD1 blockade pathway (Fig 3R and 3S), revealing that ZNF503-AS2 may be involved in immunotherapy. We also assessed the relationship between ZNF503-AS2 and MHC, chemokines, and chemokine receptors, and the results showed an association between ZNF503-AS2 and these genes (S7A–S7C Fig).

### ZNF503-AS2 is associated with the activation of multiple pathways

**Fig 4:**
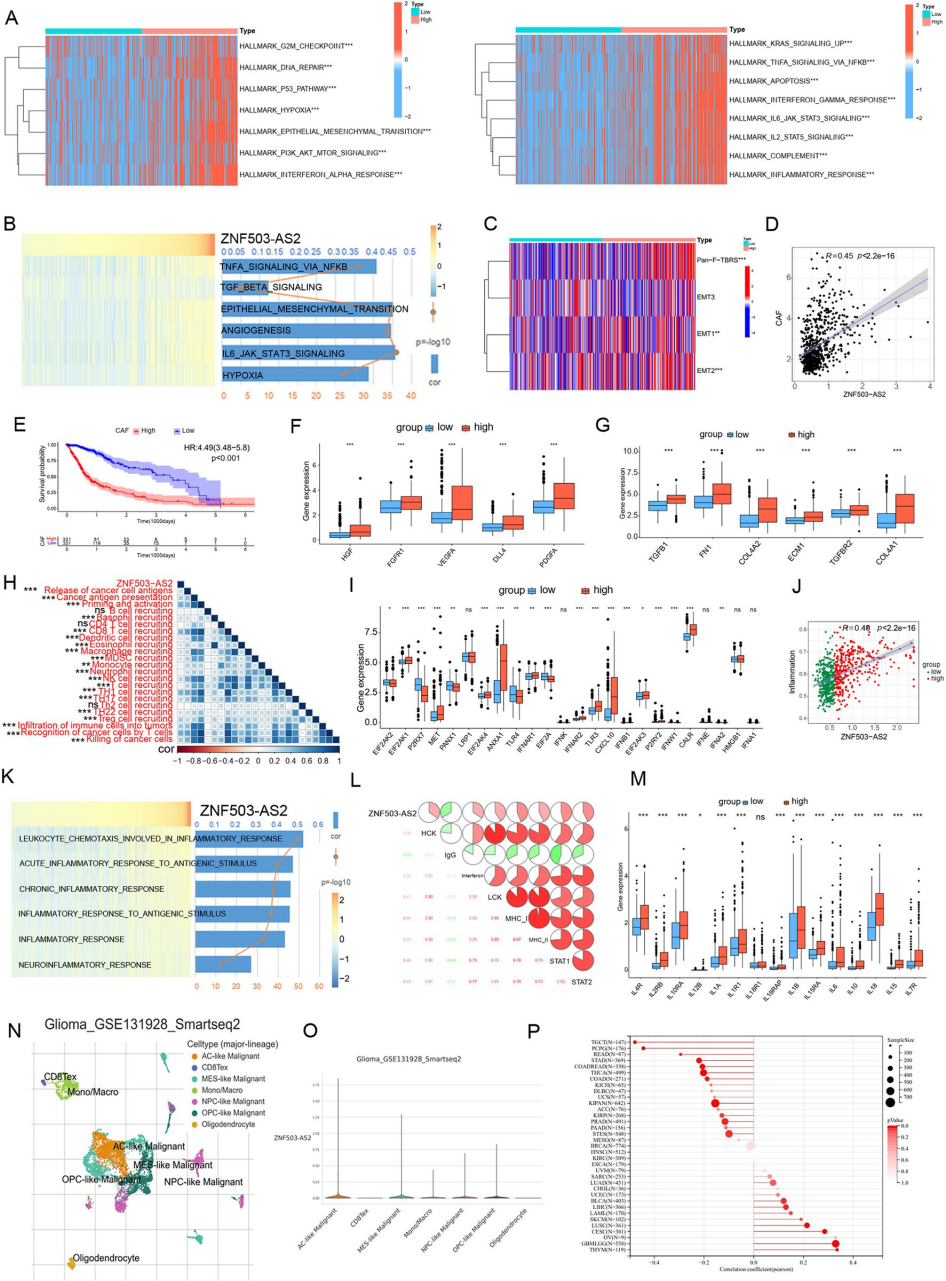
Effect of ZNF503-AS2 on the biological behavior of glioma. (A) Multiple pathways were activated in the ZNF503-AS2 high-expression group. (B) Correlation between ZNF503-AS2 and the stromal activation pathway. (C) Differential analysis of stromal activation pathways between the two groups. (D) Correlation analysis between fibroblast enrichment and ZNF503-AS2. (E) Survival analysis shows poorer OS in patients with higher fibroblast enrichment scores. (F) Differential expression of angiogenic markers and proangiogenic factors between the two groups. (G) Differential expression of key molecules affecting lymphocyte infiltration between the two groups. (H) ZNF503-AS2 was positively correlated with multiple cancer immune cycle steps. (I) Differential expression of ICD-related genes. (J) Enriched scores of inflammatory responses were positively correlated with ZNF503-AS2. (K) Correlation analysis of multiple inflammatory processes with ZNF503-AS2. (L) Correlation of ZNF503-AS2 with inflammatory-related metagenes. (M) Multiple inflammatory factors are upregulated in the high ZNF503-AS2 expression group. (N, O) Analysis of the single-cell dataset GSE131928 showed that ZNF503-AS2 was associated with malignant progression of glioma. Single-cell data and images were obtained from TISCH2. (P) Pearson correlation analysis showed that ZNF503-AS2 was positively correlated with the cancer stemness index in glioma. NS, not statistically significant; * *P* < 0.05; ** *P* < 0.01; *** *P* < 0.001.

To investigate the impact of ZNF503-AS2 on the biological behavior of glioma, we performed enrichment analysis of glioma samples from the TCGA database (Fig 4A), which showed that pathways associated with carcinogenesis and stromal pathways were activated. Stromal cells, as an important component of TME, are involved in tumor progression and suppression of T-cell function, and high expression of ZNF503-AS2 promoted the activation of the stromal pathway (Fig 4B and 4C) and positively correlated with the enrichment of cancer-associated fibroblast (CAF) (Fig 4D), which are a type of stromal cell, and high fibroblast levels predicted shorter OS than low fibroblast levels (Fig 4E). Angiogenic marker (FGFR1, DLL4, VEGFA) and proangiogenic factor (PDGFA, HGF) expression was upregulated in the high-expression group (Fig 4F), and the expression of key molecules forming the barrier to lymphocyte infiltration was higher in the group with high expression of ZNF503-AS2, which may promote the formation of an inhibitory immune microenvironment (Fig 4G). There was also a positive correlation between ZNF503-AS2 and most of the steps in the cancer immune cycle (Fig 4H), which may be helpful for improving antitumor immunity, and immunogenic cell death (ICD)-related genes associated with immunotherapy were also differentially expressed between the two groups (Fig 4I). Activation of the inflammatory response influences the TME and prognosis. Based on our analysis results, we found a positive correlation between ZNF503-AS2 and the enrichment score of the inflammatory response (Fig 4J and 4K), and the expression of ZNF503-AS2 was positively correlated with HCK, interferon, LCK, MHC-I, MHC-II, STAT1 and STAT2 (Fig 4L). Key factors involved in the inflammatory response were also significantly different between the two groups (Fig 4M). Subsequent analysis of the single-cell dataset GSE131928 demonstrated that ZNF503-AS2 expression was mainly concentrated in malignant glioma (Fig 4N and 4O) and that ZNF503-AS2 was positively correlated with the cancer stemness index of glioma (R = 0.33, *P* < 0.001), suggesting that ZNF503-AS2 may be involved in the malignant progression of glioma (Fig 4P). To increase the reliability of the results, we validated the above findings in the CGGA325 and CGGA693 databases, which showed that ZNF503-AS2 may promote the activation of the oncogenic pathway, the stromal pathway, and the inflammatory response, which influences the biological behavior of glioma and contributes to the progression (S8 Fig).

### ZNF503-AS2 is associated with methylation, mismatch repair genes, RNA modification genes and TMB

**Fig 5:**
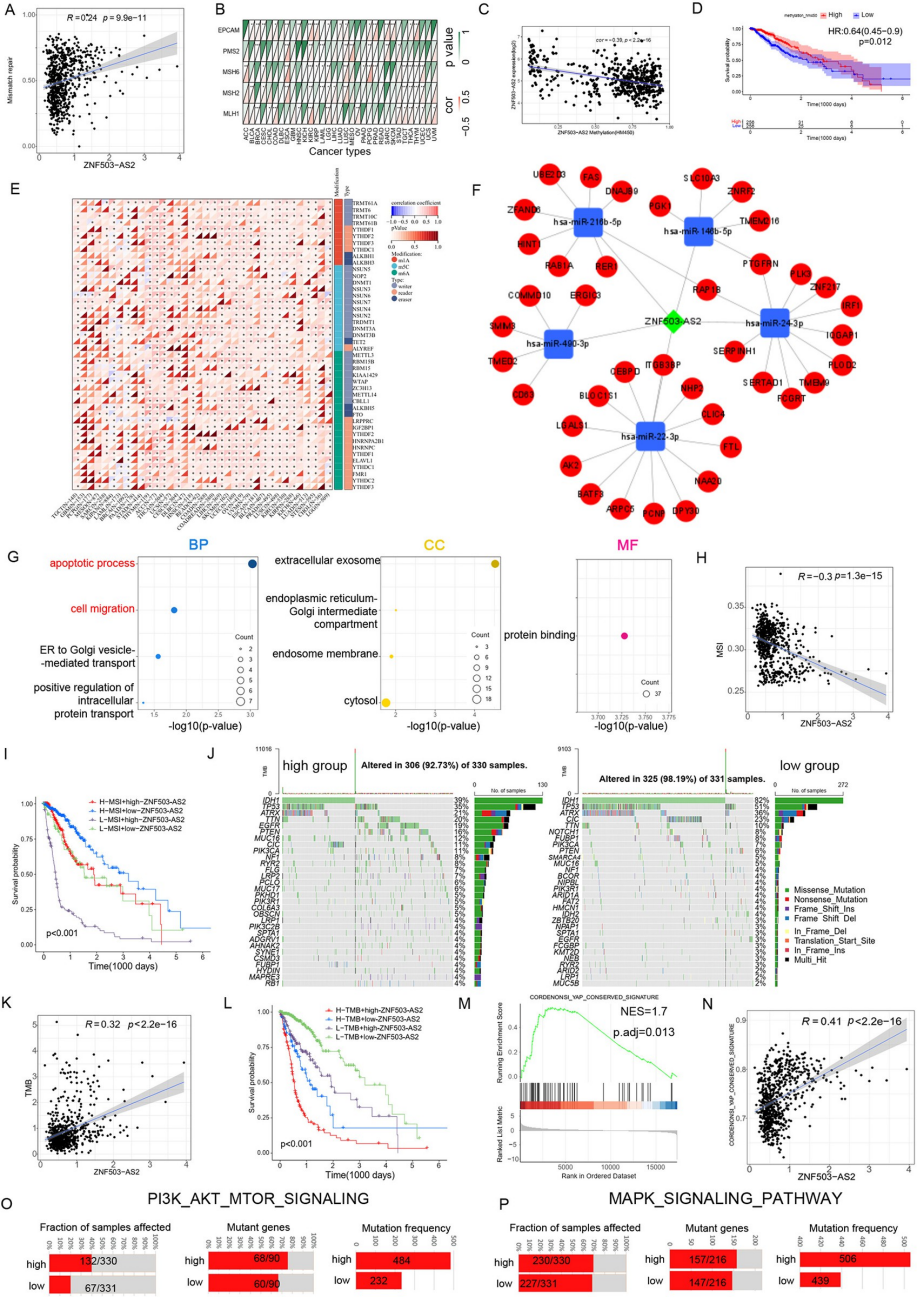
ZNF503-AS2 is associated with mismatch repair genes, methylation, MSI and TMB. (A) ZNF503-AS2 is positively correlated with the enrichment score of the mismatch repair gene set. (B) ZNF503-AS2 was significantly correlated with four mismatch repair genes in LGG. (C) Negative correlation between ZNF503-AS2 methylation level and expression. (D) Significant OS advantage in patients with high ZNF503-AS2 methylation levels. (E) Heatmap of the correlation between ZNF503-AS2 and modification genes. (F) ceRNA network constructed by ZNF503-AS2, 5 miRNAs and 40 mRNAs. (G) GO enrichment analysis of 40 mRNAs. (H) ZNF503-AS2 was negatively correlated with MSI. (I) Survival analysis of glioma patients by ZNF503-AS2 expression combined with MSI. (J) Waterfall plot showing somatic mutations in the two groups. (K) ZNF503-AS2 showed a positive correlation with TMB. (L) Combined TMB and ZNF503-AS2 expression for survival analysis. (M, N) GSEA (M) and correlation analysis (N) showed a correlation between ZNF503-AS2 and the YAP1 signaling pathway. (O, P) Distribution of mutations in the PI3K-AKT-MTOR signaling pathway (O) and MAPK signaling pathway (P) in the ZNF503-AS2 high-expression group and ZNF503-AS2 low-expression group. NS, not statistically significant; * *P* < 0.05; ** *P* < 0.01; *** *P* < 0.001.

After predicting the function of ZNF503-AS2, we investigated the association between ZNF503-AS2 and epigenetic modifications. ZNF503-AS2 was positively correlated with the enrichment scores of mismatch repair genes (Fig 5A) and ZNF503-AS2 was significantly correlated with four mismatch repair genes in LGG (Fig 5B). We found that the methylation degree of ZNF503-AS2 was negatively correlated with its expression (Fig 5C) and that patients with low ZNF503-AS2 methylation had a poorer prognosis (Fig 5D). In addition, ZNF503-AS2 was closely associated with RNA modification genes in LGG (Fig 5E). To further investigate how ZNF503-AS2 regulates mRNA expression, we screened miRNAs interacting with ZNF503-AS2 from the miRcode database and searched for target mRNAs of these miRNAs. Because lncRNAs mostly elevate mRNA expression by acting as ceRNAs, we screened 40 mRNAs with the strongest correlation with ZNF503-AS2 among these target mRNAs. Finally, we constructed a ceRNA network including ZNF503-AS2, miRNA and mRNA (Fig 5F). We performed functional enrichment analysis for these 40 mRNAs, and GO analysis showed that the apoptotic process, cell migration and other processes were enriched, MF was extracellular exosome, and CC was protein binding (Fig 5G).

The combined analysis of ZNF503-AS2 with microsatellite instability (MSI) and TMB is beneficial for better prediction of immunotherapy efficacy and patient prognosis. In other cancers, MSI-H patients had a greater chance of benefiting from immunotherapy, and ZNF503-AS2 was negatively correlated with MSI in glioma (Fig 5H). The combined survival analysis of MSI and ZNF503-AS2 showed that the H-MSI and low ZNF503-AS2 expression groups had the best prognosis (Fig 5I). The waterfall plot showed that IDH, TP53, and ATRX mutations, which are considered to be LGG markers, were mainly distributed in the low-expression group (Fig 5J), and ZNF503-AS2 was positively correlated with TMB (Fig 5K). Patients with high TMB and high ZNF503-AS2 expression had a worse prognosis (Fig 5L). According to previous reports, PTEN deficiency activates the YAP1 signaling pathway and increases macrophage infiltration, which in turn secretes SPP1 to promote the growth and survival of GBM and the formation of an inhibitory immune microenvironment [30]. GSEA showed that the YAP1 signaling pathway was upregulated in the high ZNF503-AS2 expression group (Fig 5M), and ZNF503-AS2 showed a correlation with the YAP signaling pathway enrichment score (Fig 5N). The same trend was observed in the CGGA325 and CGGA693 datasets (S9A and S9B Fig). In addition, our analysis of signaling pathways revealed that the PI3K-AKT-MTOR signaling pathway and MAPK signaling pathway were the carcinogenic pathways with the largest difference in mutation frequency between the two groups (Fig 5O and 5P).

### Analysis of the potential value of ZNF503-AS2 in glioma therapy

**Fig 6:**
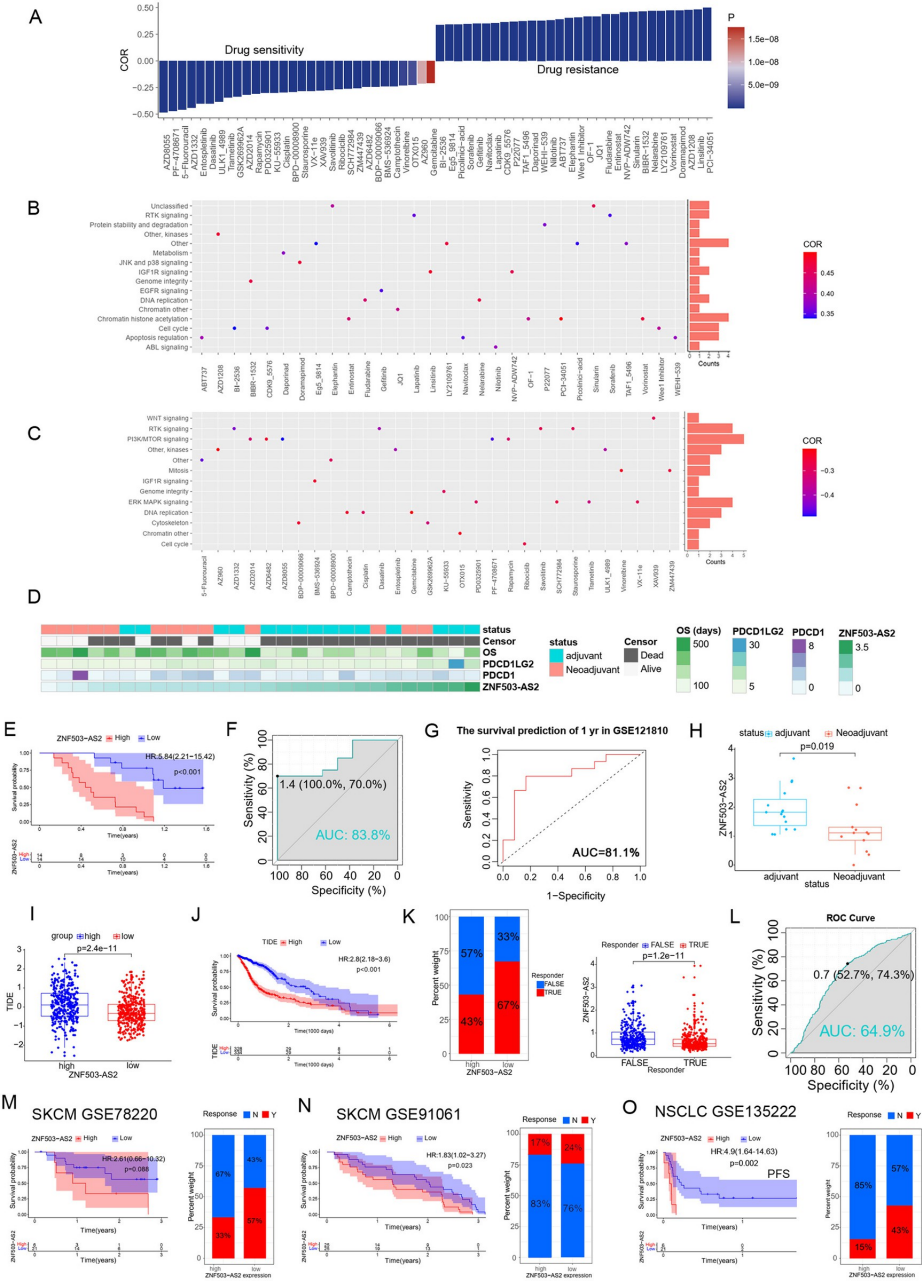
ZNF503-AS2 had predictive value for chemotherapy and immunotherapy in glioma. (A) Ranking of the most sensitive and most resistant drugs with ZNF503-AS2 expression. (B, C) Signaling pathways targeted by the most resistant (B) and most sensitive (C) drugs with ZNF503-AS2 expression. (D) Distribution of clinical characteristics and ZNF503-AS2 expression in patients from the GSE121810 database. (E) GBM patients with high ZNF503-AS2 expression receiving immunotherapy have a poorer prognosis. (F, G) ROC curves showed that ZNF503-AS2 was highly accurate in predicting OS (F) and 1-year OS (G) in GBM patients receiving immunotherapy. (H) Decreased expression of ZNF503-AS2 in patients treated with neoadjuvant immunotherapy compared to that in patients treated with immunotherapy alone. (I) Higher TIDE scores in the high ZNF503-AS2 expression group. (J) Patients with high TIDE scores had a worse prognosis. (K) The low ZNF503-AS2 expression group may have a better response to immunotherapy. (L) Value of ZNF503-AS2 in the prediction of response to immunotherapy. (M-O) ZNF503-AS2 was a prognostic risk factor in SKCM (M, N) and NSCLC (O), and patients with low ZNF503-AS2 expression responded better to immunotherapy. NS, not statistically significant; * *P* < 0.05; ** *P* < 0.01; *** *P* < 0.001.

To investigate the guiding role of ZNF503-AS2 in drug selection, we screened 154 drugs associated with ZNF503-AS2 through the GDSC database. Based on the correlation between the half-maximal inhibitory concentration (IC50) of drugs and ZNF503-AS2 expression, we listed the 30 most sensitive drugs and the 30 most resistant drugs for glioma patients with high ZNF503-AS2 expression (Fig 6A). After analyzing the pathways targeted by these drugs, we found that drugs resistant to ZNF503-AS2 expression primarily target chromatin histone acetylation (Fig 6B), and the most sensitive drugs mainly targeted the PI3K/MTOR signaling pathway and ERK MAPK signaling pathway (Fig 6C). In addition, we also evaluated the relationship between the sensitivity of commonly used chemotherapeutic drugs and ZNF503-AS2, and the results showed that patients with high ZNF503-AS2 expression were more sensitive to camptothecin, cisplatin, crizotinib, entospletinib, rapamycin, talazoparib, topotecan, and trametinib (S9C–S9J Fig). These results suggest that multiple chemotherapeutic drugs may have better efficacy in patients with high ZNF503-AS2 expression, which may be helpful for drug selection in glioma. In addition, we analyzed the effect of ZNF503-AS2 on OS in patients receiving temozolomide chemotherapy. Among patients receiving temozolomide chemotherapy, those with high ZNF503-AS2 expression had a worse prognosis (S9K Fig), and we found that whether the MGMT promoter was methylated did not affect this finding (S9L and S9M Fig). For patients receiving radiotherapy, patients with low ZNF503-AS2 expression had a significant benefit (S9N Fig). Immunotherapy has gained widespread attention as an advanced, effective treatment option for many cancers. However, its efficacy in glioma is not yet satisfactory, partially due to the lack of effective biomarkers that can evaluate the efficacy of immunotherapy. We evaluated the role of ZNF503-AS2 in the efficacy of neoadjuvant anti-PD-1 immunotherapy for recurrent GBM using the GSE121810 database. The clinical landscape of the 28 glioma patients who received immunotherapy is shown in Fig 6D. The analysis showed that the survival of patients with low ZNF503-AS2 expression was prolonged after immunotherapy (Fig 6E), and the ROC curves indicated that ZNF503-AS2 had a better predictive ability for OS (AUC = 0.838; Fig 6F) and 1-year OS (AUC = 0.811; Fig 6G). We also found that the expression of ZNF503-AS2 was decreased in patients receiving neoadjuvant immunotherapy compared to that in the patients receiving immunotherapy alone (Fig 6H), which may offer a new strategy for patients with poor treatment outcomes. In addition, we validated the role of ZNF503-AS2 in glioma immunotherapy using the TIDE tool. The TIDE score was higher in the high ZNF503-AS2 expression group (Fig 6I), while the higher TIDE score was associated with a greater likelihood of immune escape, and the survival analysis revealed a survival disadvantage in patients with high TIDE score groups (Fig 6J). We also found that most patients who responded to immunotherapy were distributed in the low-expression group (Fig 6K), and ZNF503-AS2 performed satisfactorily in predicting immunotherapy efficacy (Fig 6L). To further validate our conclusions, we further evaluated the role of ZNF503-AS2 in immunotherapy using other datasets of cancers receiving immunotherapy, which showed that patients with high expression of ZNF503-AS2 benefited less from immunotherapy and might have a poorer response to receiving immunotherapy, which is a consistent trend with the results observed in patients with glioma (Fig 6M–6O). However, the role of ZNF503-AS2 in immunotherapy for other cancers still needed further investigation.

### Knockdown of ZNF503-AS2 decreases GBM cell proliferation, leads to G2/M cell cycle arrest, promotes apoptosis, and reduces cell invasion and migration capacity

**Fig 7:**
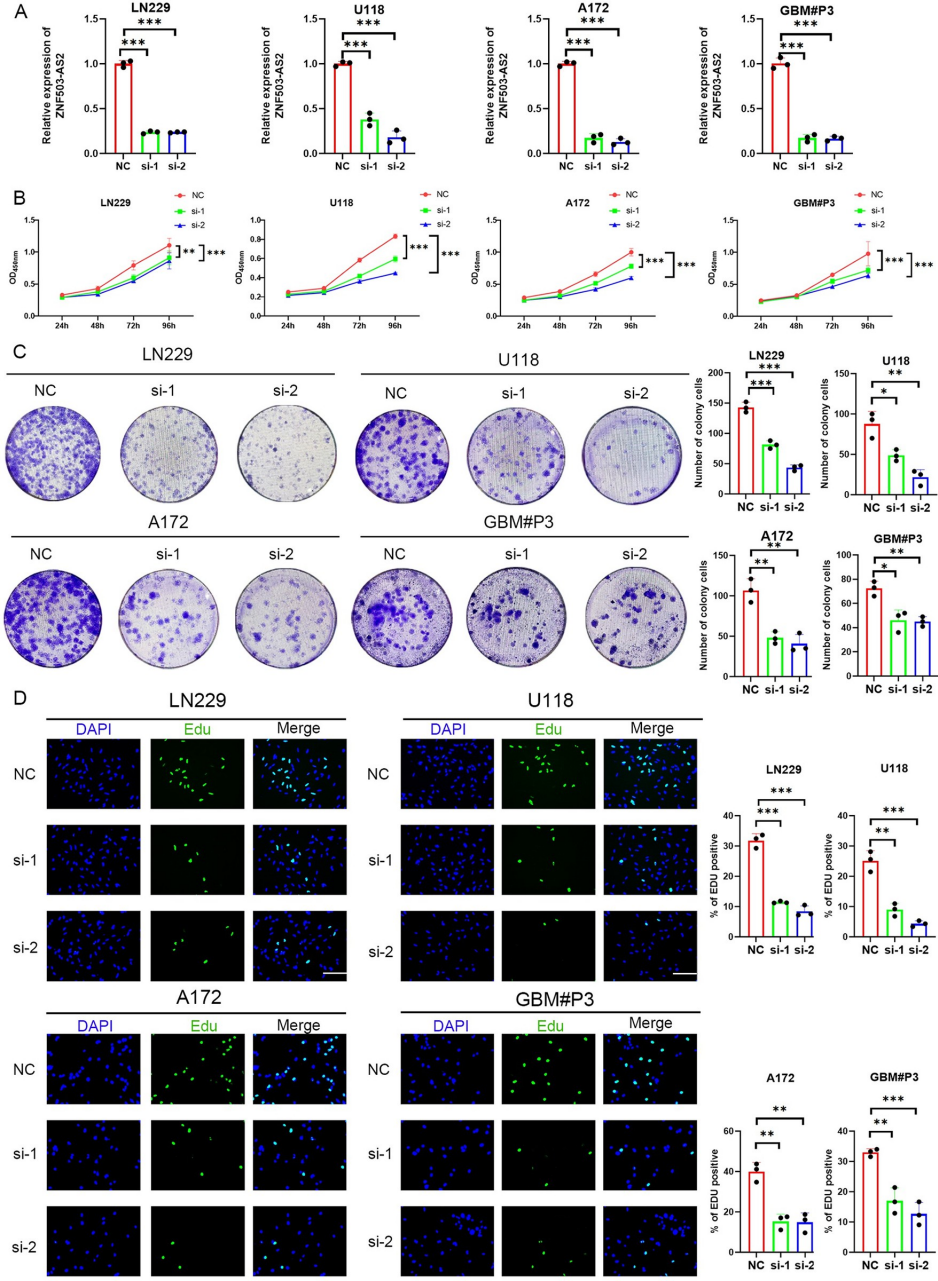
Knockdown of ZNF503-AS2 inhibited cell proliferation. (A) The knockdown efficiency of ZNF503-AS2 was measured by qRT‒PCR. (B-D) CCK-8 (B), colony formation assay (C), and EdU assay (D) showed that ZNF503-AS2 knockdown reduced the proliferative capacity of LN229, U118, A172, and GBM#P3 cells. Scale bar = 100 μm. NS, not statistically significant; * *P* < 0.05; ** *P* < 0.01; *** *P* < 0.001.

To evaluate the role of ZNF503-AS2 in GBM cells, we knocked down ZNF503-AS2 in GBM cell lines (LN229, U118 and A172) and primary GSCs (GBM#P3) using siRNAs, and the knockdown efficiency is shown in Fig 7A. The results of the CCK-8, colony formation assay, and EdU assay showed that the growth of ZNF503-AS2 knockdown cells was inhibited (Fig 7B–7D).

**Fig 8:**
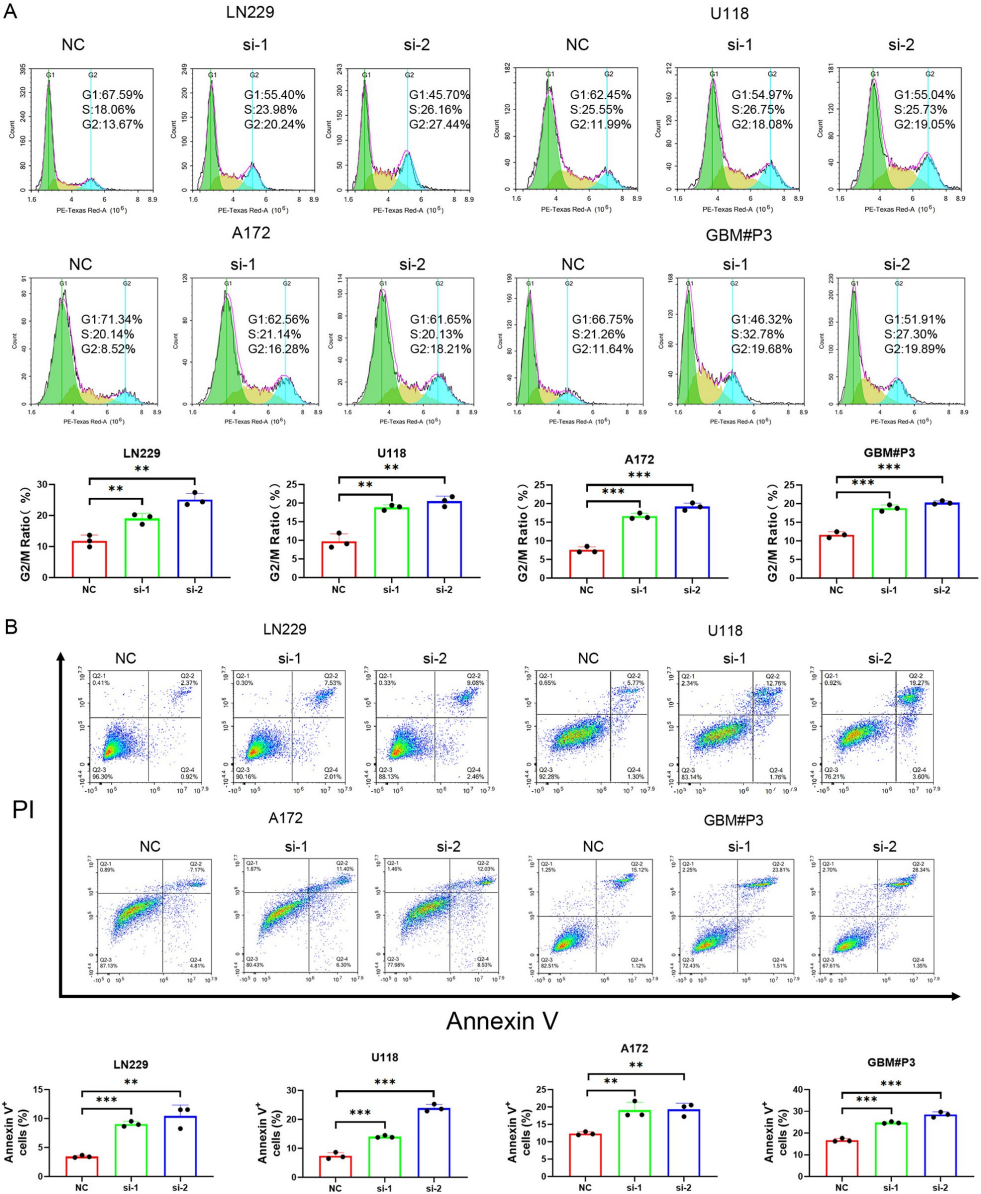
Detection of the cell cycle and apoptosis by flow cytometry. (A) ZNF503-AS2 knockdown resulted in an increase in G2/M phase cells. (B) Knockdown of ZNF503-AS2 increased apoptosis in LN229, U118, A172, and GBM#P3 cells. NS, not statistically significant; * *P* < 0.05; ** *P* < 0.01; *** *P* < 0.001.

Analysis of the cell cycle assay showed that knockdown of ZNF503-AS2 led to an increase in G2/M phase cells (Fig 8A), and the results of apoptosis showed that ZNF503-AS2 knockdown was able to promote apoptosis (Fig 8B).

**Fig 9:**
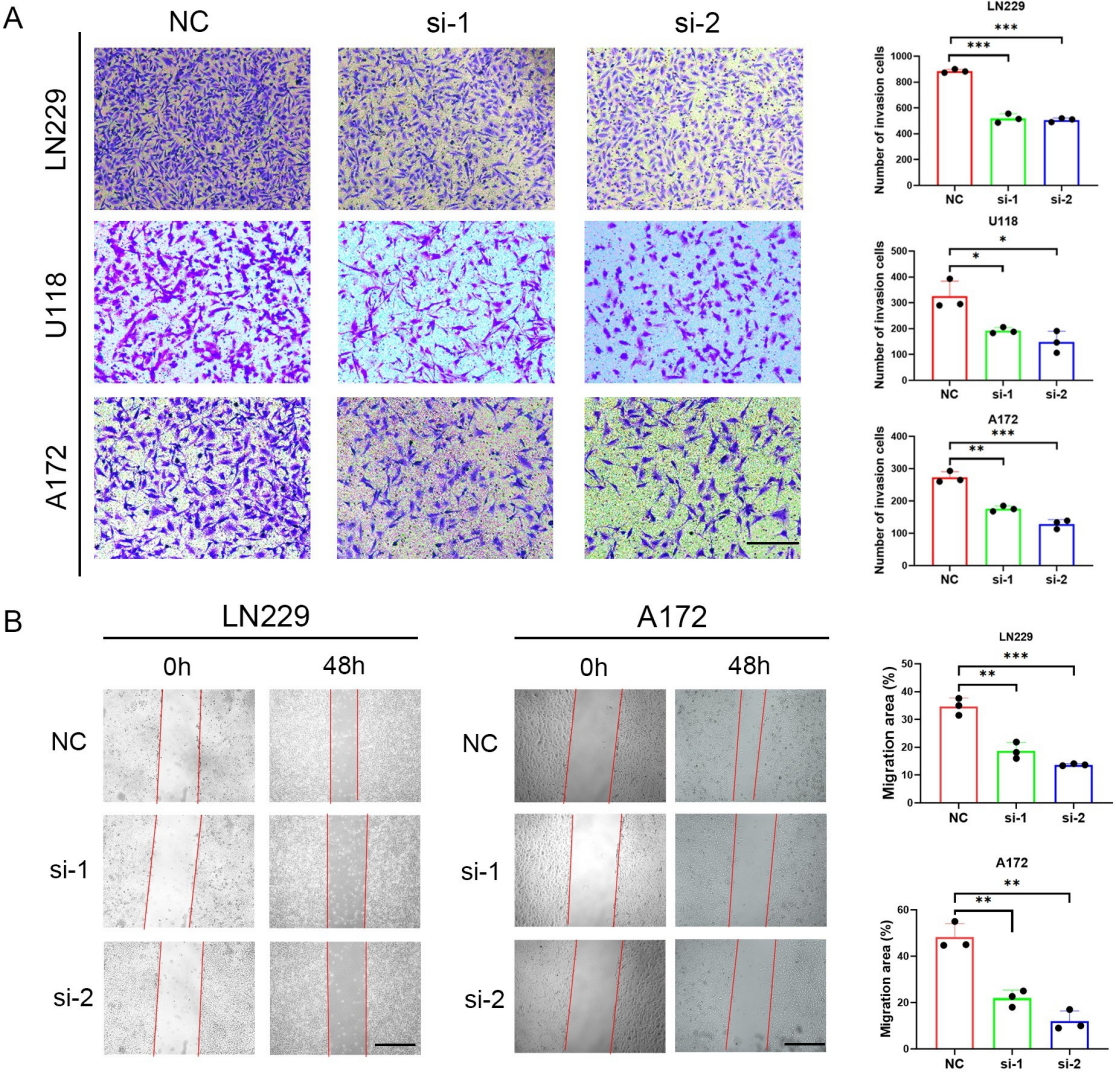
Knockdown of ZNF503-AS2 inhibits invasion and migration. (A) Transwell assay in LN229, U118 and A172 transfected with ZNF503-AS2 siRNAs or NC. Scale bar = 200 μm. (B) Wound healing assay in LN229 and A172 cells transfected with ZNF503-AS2 siRNAs or NC. Scale bar = 200 μm. NS, not statistically significant; * *P* < 0.05; ** *P* < 0.01; *** *P* < 0.001.

The results of Transwell assays and wound healing assays showed that ZNF503-AS2 knockdown inhibited cell invasion (Fig 9A) and migration (Fig 9B).

### Comprehensive analysis of the role of ZNF503-AS2 in pancancer

We found that the expression of ZNF503-AS2 was significantly different in all cancers except uterine corpus endometrial carcinoma (UCEC), cervical squamous cell carcinoma and endocervical adenocarcinoma (CESC), testicular germ cell tumor (TGCT), adrenocortical carcinoma (ACC), and kidney chromophobe carcinoma (KICH) by analyzing the TCGA combined with the GTEx and TCGA databases (S10A and S10B Fig), and we found that in colon adenocarcinoma (COAD), kidney renal clear cell carcinoma (KIRC), pancreatic adenocarcinoma (PAAD), and thyroid carcinoma (THCA), ZNF503-AS2 expression was closely related to tumor stage (S10C Fig). Moreover, correlation analysis showed that TMB was correlated with 13 tumors, MSI was correlated with 9 tumors, stromal score showed a correlation with 18 tumors, immune score showed a correlation with 19 tumors (S10D–S10G Fig), and ESTIMATE score and TumorPurity were associated with 17 cancers (S10H and S10I Fig). We found that ZNF503-AS2 was associated with the prognosis of many tumors (S11 and S12 Figs). ZNF503-AS2 was associated with immune cell infiltration in all cancers except uveal melanoma (UVM) and cholangiocarcinoma (CHOL, S13A Fig). ZNF503-AS2 was significantly correlated with the stemness index in several tumors (S13B Fig). In addition, we found that in glioma, ZNF503-AS2 was significantly associated with LOH (R = 0.29, *P* < 0.001), MATH (R = -0.35, *P* < 0.001) and tumor ploidy (R = -0.094, *P* < 0.05), but not with HRD (ns) (S13C–S13F Fig).

## Discussion

Glioma is the most common intracranial malignant tumor, and there are no treatments that can effectively improve its prognosis. In the last decade, immunotherapy, as a promising therapeutic approach, has not been satisfactory in treating glioma [31,32], but its breakthroughs in many other cancers have been encouraging for the treatment of glioma [33,34], so exploring the mechanisms underlying immunotherapy and searching for new and effective biologic markers has become a novel method for improving the therapeutic outcome [35].

In this study, we analyzed the TCGA and CGGA databases and determined that ZNF503-AS2 expression was significantly elevated in glioma and was associated with poor prognosis. Enrichment analysis showed that the DEGs were significantly enriched in processes related to tumor immunity in addition to the oncogenic pathway, possibly suggesting that ZNF503-AS2 participates in glioma TME remodeling. The complex immune microenvironment of glioma has been shown to be associated with poor immunotherapeutic efficacy [36]; therefore, we focused on the effect of ZNF503-AS2 on the TME. First, we found that ZNF503-AS2 was most highly expressed in MES subtypes, which have been found to be enriched with immunosuppressive cells in the microenvironment in previous studies [37], and this conclusion was supported by an analysis of immune cell infiltration. We found that immunosuppressive cell (macrophages, neutrophils, Tregs, MDSCs) infiltration was increased in the ZNF503-AS2 high-expression group and produced large amounts of chemokines, which may be interpreted as a contributing factor to the poor response to immunotherapy [38,39]. Analysis of single-cell datasets supported our conclusion that ZNF503-AS2 is expressed mainly in tumor cells and macrophages, and survival analyses showed that the effect of ZNF503-AS2 on OS was not dependent on macrophage infiltration; therefore, targeting ZNF503-AS2 may be effective in patients with high macrophage infiltration. In addition, ZNF503-AS2 was positively correlated with a variety of immune checkpoints, whose high expression promoted immunosuppression and ultimately led to immune escape, and we found that the DEGs were also significantly enriched in the PD1 pathway and immunotherapy by the PD1 blockade pathway, revealing a possible link between ZNF503-AS2 and immunotherapy. Based on the functional analysis of ZNF503-AS2, we found that ZNF503-AS2 promoted the activation of oncogenic pathways, stroma-associated pathways, and inflammatory responses, and the activation of stromal cells and production of inflammatory factors further altered the TME, which showed that ZNF503-AS2 could affect the glioma TME in various ways [40,41].

We also evaluated the correlation between ZNF503-AS2 and TMB and MSI, the predictors of immunotherapy efficacy, and survival analysis also revealed that the combined analysis of ZNF503-AS2 with TMB and MSI could better assess the prognosis of patients. The waterfall plot revealed a decrease in IDH mutations, ATRX mutations, and CIC mutations in the high ZNF503-AS2 expression group and an increase in PTEN mutations, which have been reported to promote TAM infiltration in previous studies. Finally, analysis of the GSE121810 dataset showed that ZNF503-AS2 was a good predictor of OS in patients receiving immunotherapy. The use of the TIDE tool further validated our conclusions, and it also predicted that patients with low ZNF503-AS2 expression may be more likely to benefit from immunotherapy. We also validated the role of ZNF503-AS2 in glioma through a series of in vitro experiments.

## Conclusions

In conclusion, this study demonstrated that ZNF503-AS2 expression is elevated in glioma and has a good predictive ability for the prognosis of glioma patients, and the knockdown of ZNF503-AS2 can inhibit the proliferation, invasion, and migration of glioma cells, induce G2/M cell cycle arrest, and promote the apoptosis of glioma cells. Functional analysis showed a close relationship between ZNF503-AS2 and tumor immunity, and further analysis revealed that ZNF503-AS2 promoted the infiltration of immunosuppressive cells and that the activation of the stromal pathway and inflammatory response promoted by ZNF503-AS2 also resulted in remodeling of the glioma TME. Finally, the excellent performance of ZNF503-AS2 in immunotherapy also showed its potential as a possible immunotherapeutic target in the future.

## Supporting information

S1 Fig Relationship between ZNF503-AS2 and clinical characteristics in the TCGA database. (A) ZNF503-AS2 expression is upregulated in GBM in the TCGA database. (B) ROC curve analysis of ZNF503-AS2 predicts GBM in the TCGA database. (C-E) ZNF503-AS2 expression is upregulated in GBM (C), LGG (D) and all glioma (E) in TCGA combined with the GTEx database. (F) ZNF503-AS2 was significantly positively correlated with the enrichment scores of the MES and CL subtypes and significantly negatively correlated with the enrichment scores of the NE and PN subtypes. (G, H) ZNF503-AS2 was positively correlated with the MES subtype markers CD44 (G) and CHI3L1 (H). (I) Heatmap of MES signature genes. NS, not statistically significant; * *P* < 0.05; ** *P* < 0.01; *** *P* < 0.001.
(TIF)

S2 Fig Relationship between ZNF503-AS2 and clinical characteristics in the CGGA325 and CGGA693 databases. (A, B) Distribution of ZNF503-AS2 in different WHO grades, IDH statuses, 1p/19q statuses, and ages in the CGGA325 (A) and CGGA693 databases (B). (C) ZNF503-AS2 expression was highest in MES subtypes in the CGGA325. (D) The MES subtype and CL subtype were mainly distributed in the ZNF503-AS2 high-expression group in the CGGA325. (E) ZNF503-AS2 was significantly positively correlated with the enrichment scores of the MES subtype and CL subtype and significantly negatively correlated with the enrichment scores of the PN and NE subtypes in the CGGA325 and CGGA693 databases. (F) Differential expression of MES and PN markers between the two groups in the CGGA325 and CGGA693 databases. (G) Correlation analysis showed that ZNF503-AS2 was significantly positively correlated with the MES subtype markers CD44 and CHI3L1 in the CGGA325 and CGGA693 databases. (H) Heatmap of MES signature genes in the CGGA325 and CGGA693 databases. (I) GSEA showed that samples with high expression of ZNF503-AS2 were enriched in the MES subtype and CL subtype in the CGGA325 and CGGA693 databases.
(TIF)

S3 Fig Value of ZNF503-AS2 in predicting clinical characteristics. (A) ROC curve to assess the value of ZNF503-AS2 for GBM prediction. (B) ROC curve to assess the value of ZNF503-AS2 for IDH status prediction. (C) ROC curve to assess the value of ZNF503-AS2 for 1p/19q codeletion prediction. (D) ROC curve to assess the value of ZNF503-AS2 expression in predicting the MES molecular subtype and CL molecular subtype.
(TIF)

S4 Fig Effect of ZNF503-AS2 on glioma prognosis and function in the CGGA database. (A, B) Univariate and multivariate COX regression analysis of clinical data and ZNF503-AS2 expression in glioma samples in the CGGA325 database (A) and CGGA693 database (B). (C, D) Time-dependent ROC curves showed high predictive accuracy of ZNF503-AS2 for OS at 1, 3, and 5 years in glioma patients in the TCGA (C) and CGGA325 databases (D). (E) Nomogram combining ZNF503-AS2 and clinical characteristics predicts patient OS. (F) Calibration plots of predicted OS and actual OS. (G) Enrichment analysis of DEGs in the CGGA325 databases. (H, I) GSEA of DEGs in GO biological processes (H) and hallmark gene sets (I) in the CGGA325 databases. (J) PaGenBase shows that the DEGs are highly specifically expressed in the blood and spleen in the CGGA325 databases. (K) TRRUST shows that DEGs are mainly regulated by SP1, NFKB1 and RELA in the CGGA325 databases.
(TIF)

S5 Fig Correlation of ZNF503-AS2 with multiple immunosuppressive cell markers. (A) Correlation of ZNF503-AS2 with markers of MDSCs. (B) Correlation of ZNF503-AS2 with markers of TAMs. (C) Correlation of ZNF503-AS2 with markers of Tregs.
(TIF)

S6 Fig ZNF503-AS2 is associated with the TME in glioma. (A, C) ssGSEA showed significant differences in immune cell and immune function enrichment scores between the two groups in the CGGA325 (A) and CGGA693 databases (C). (B, D) Correlation analysis of stromal score, immune score, ESTIMATE score, and tumor purity with ZNF503-AS2 in the CGGA325 (B) and CGGA693 databases (D). (E, F) Correlation analysis of immunosuppressive cells with ZNF503-AS2 in the CGGA325 (E) and CGGA693 databases (F). (G) Correlation analysis of two TAMs with ZNF503-AS2 in the CGGA325 and CGGA693 databases. (H) ZNF503-AS2 is significantly and positively correlated with immune checkpoint and HLA enrichment scores in the CGGA325 and CGGA693 databases. (I) Heatmap of the correlation between chemotaxis of immunosuppressed cells and ZNF503-AS2 expression in the CGGA325 and CGGA693 databases.
(TIF)

S7 Fig Correlation analysis of MHC molecules (A), chemokines (B), chemokine receptors (C) and ZNF503-AS2 expression. NS, not statistically significant; * *P* < 0.05; ** *P* < 0.01; *** *P* < 0.001.
(TIF)

S8 Fig Effect of ZNF503-AS2 on the biological behavior of glioma. (A, C) Correlation between ZNF503-AS2 and the stromal activation pathway in the CGGA325 (A) and CGGA693 databases (C). (B, D) Multiple pathways were activated in the ZNF503-AS2 high-expression group in the CGGA325 (B) and CGGA693 databases (D). (E, H) Differential expression of angiogenic markers and pro-angiogenic factors between the two groups in the CGGA325 (E) and CGGA693 databases (H). (F, I) Differential expression of key molecules affecting lymphocyte infiltration between the two groups in the CGGA325 (F) and CGGA693 databases (I). (G, J) Enriched scores of inflammatory responses were significantly positively correlated with ZNF503-AS2 in the CGGA325 (G) and CGGA693 databases (J). (K, N) Correlation analysis of multiple inflammatory processes with ZNF503-AS2 in the CGGA325 (K) and CGGA693 databases (N). (L, O) Correlation analysis of multiple inflammatory processes with ZNF503-AS2 in the CGGA325 (L) and CGGA693 databases (O). (M, P) Multiple inflammatory factors were significantly different between the two groups in the CGGA325 (M) and CGGA693 databases (P). (Q) Differential expression of ICD-related genes.
(TIF)

S9 Fig ZNF503-AS2 is promising for guiding clinical drug selection. (A, B) ZNF503-AS2 high-expression samples were enriched in the YAP1 signaling pathway and positively correlated with the enrichment score of the YAP1 signaling pathway. (C-J) Multiple drugs are sensitive to patients with high ZNF503-AS2 expression. (K) Survival analysis of patients treated with temozolomide. (L, M) Survival analysis of temozolomide-treated patients according to whether the MGMT promoter is methylated (L) or not (M). (N) Survival analysis of patients receiving radiotherapy.
(TIF)

S10 Fig Analysis of ZNF503-AS2 performance in pan-cancer. (A) ZNF503-AS2 expression in TCGA combined with GTEx. (B) ZNF503-AS2 expression in TCGA. (C) Relationship between ZNF503-AS2 expression and stage. (D-I) Correlation analysis between ZNF503-AS2 and TMB (D), MSI (E), stromal score (F), immune score (G), ESTIMATE score (H), and tumor purity (I).
(TIF)

S11 Fig Univariate Cox regression analyses of OS (A), DFS (B), DSS (C) and PFS (D).
(TIF)

S12 Fig Survival analysis in pancancer. (TIF)

S13 Fig Correlation of ZNF503-AS2 with immune cell infiltration (A), cancer stemness index (B), HRD (C), LOH (D), MATH (E) and ploidy (F).
(TIF)

## Abbreviations

lncRNAs: Long non-coding RNAs
TME: tumor microenvironment
ICB: immune checkpoint blockade
TCGA: The Cancer Genome Atlas
CGGA: Chinese Glioma Genome Atlas
OS: overall survival
LGG: lower-grade glioma
GBM: glioblastoma
GTEx: Genotype-Tissue Expression Project
TMB: Tumor mutation burden
GEO: Gene Expression Omnibus
WHO: World Health Organization
DEG: Differential expression gene
GSVA: Gene set variation analysis
MSI: microsatellite instability
