# Supplementary figures and images for: ZNF503-AS2 is a promising therapeutic target and is associated with the immune microenvironment in glioma
Source: PLoS One. 2024 Dec 2;19(12):e0314618. doi: 10.1371/journal.pone.0314618 (PMC11611154; doi:10.1371/journal.pone.0314618)

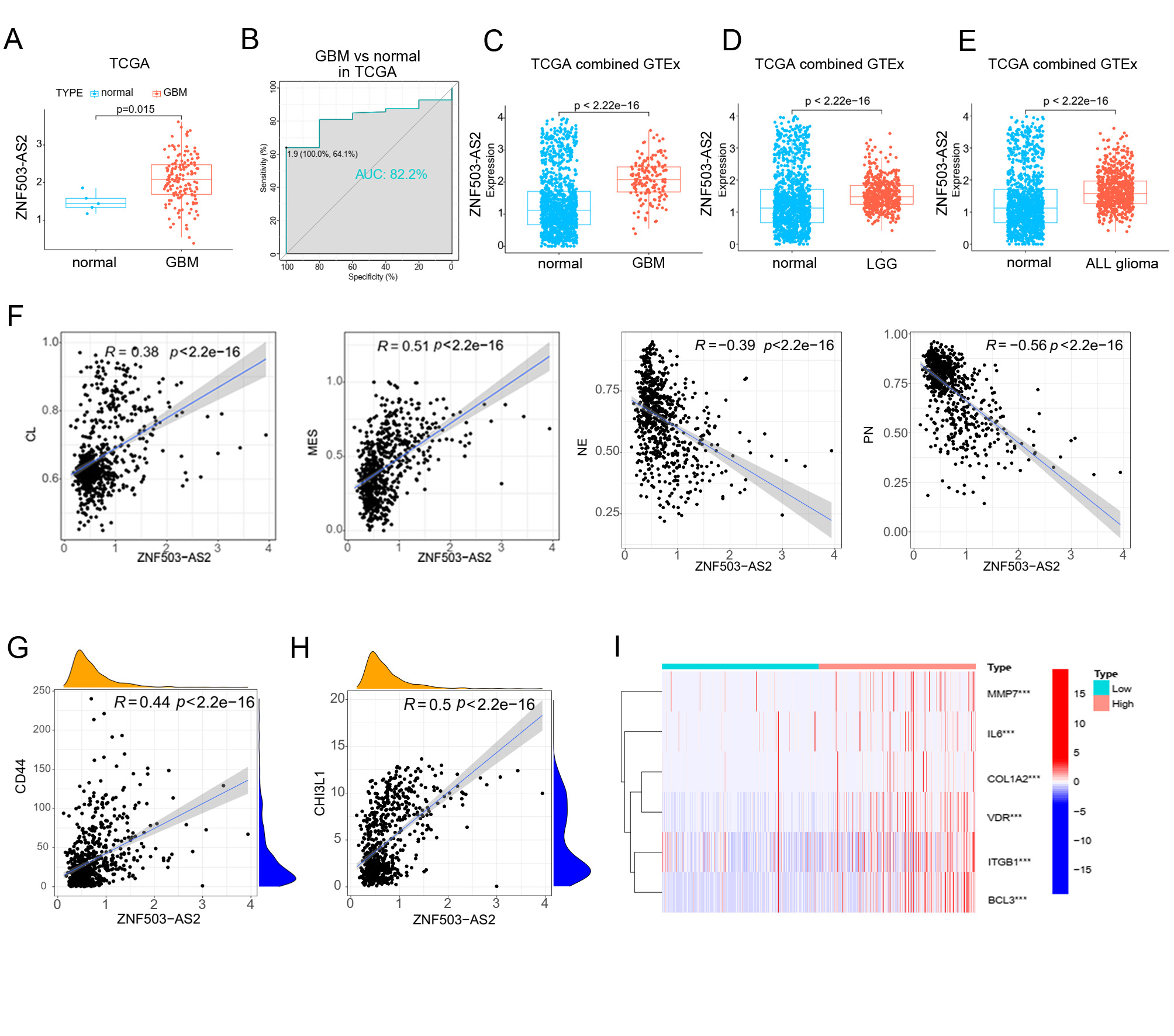

Supplement: S1 Fig — (A) ZNF503-AS2 expression is upregulated in GBM in the TCGA database. (B) ROC curve analysis of ZNF503-AS2 predicts GBM in the TCGA database. (C-E) ZNF503-AS2 expression is upregulated in GBM (C), LGG (D) and all glioma (E) in TCGA combined with the GTEx database. (F) ZNF503-AS2 was significantly positively correlated with the enrichment scores of the MES and CL subtypes and significantly negatively correlated with the enrichment scores of the NE and PN subtypes. (G, H) ZNF503-AS2 was positively correlated with the MES subtype markers CD44 (G) and CHI3L1 (H). (I) Heatmap of MES signature genes. NS, not statistically significant; * P < 0.05; ** P < 0.01; *** P < 0.001. (TIF) [file pone.0314618.s001.tif]

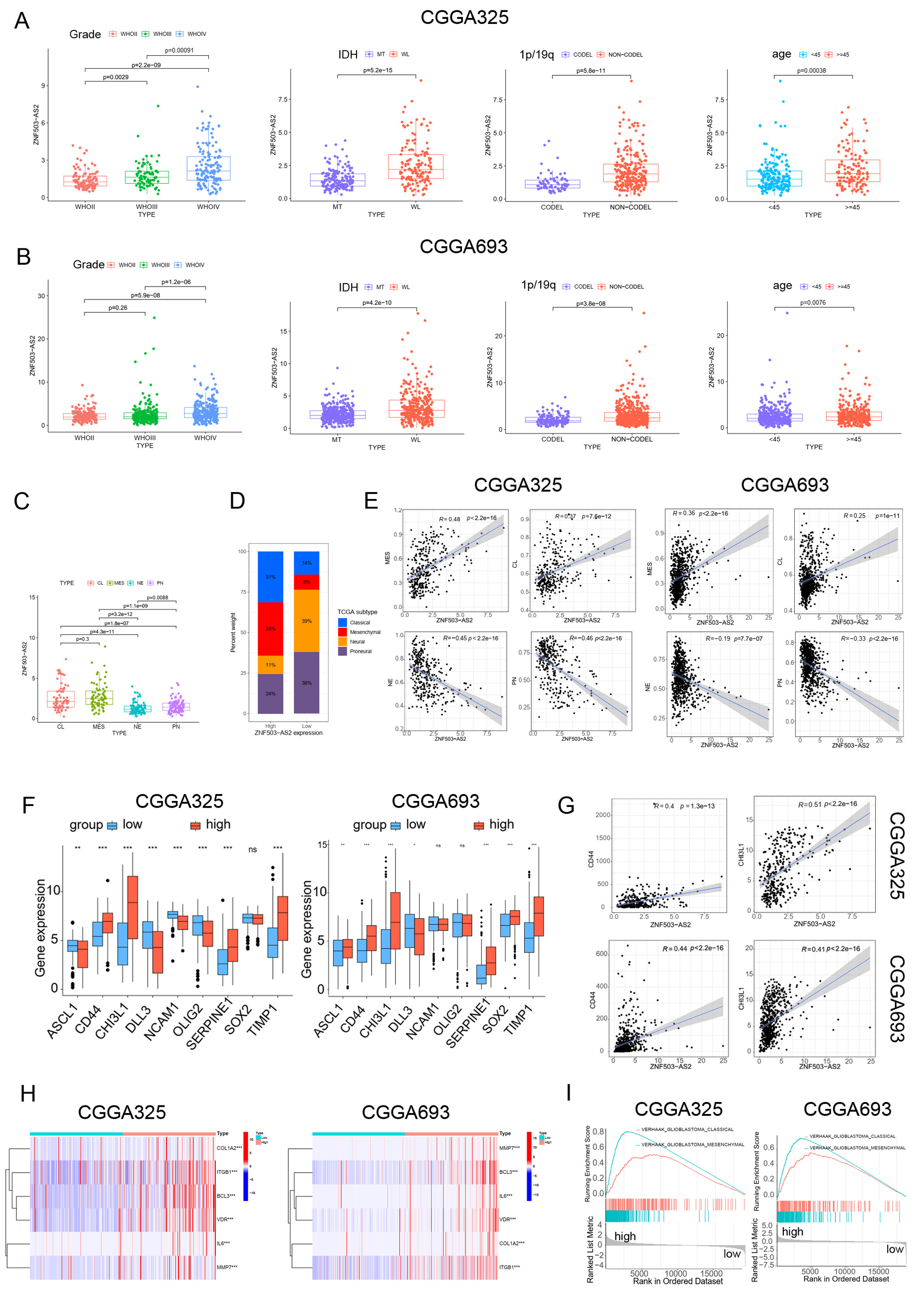

Supplement: S2 Fig — (A, B) Distribution of ZNF503-AS2 in different WHO grades, IDH statuses, 1p/19q statuses, and ages in the CGGA325 (A) and CGGA693 databases (B). (C) ZNF503-AS2 expression was highest in MES subtypes in the CGGA325. (D) The MES subtype and CL subtype were mainly distributed in the ZNF503-AS2 high-expression group in the CGGA325. (E) ZNF503-AS2 was significantly positively correlated with the enrichment scores of the MES subtype and CL subtype and significantly negatively correlated with the enrichment scores of the PN and NE subtypes in the CGGA325 and CGGA693 databases. (F) Differential expression of MES and PN markers between the two groups in the CGGA325 and CGGA693 databases. (G) Correlation analysis showed that ZNF503-AS2 was significantly positively correlated with the MES subtype markers CD44 and CHI3L1 in the CGGA325 and CGGA693 databases. (H) Heatmap of MES signature genes in the CGGA325 and CGGA693 databases. (I) GSEA showed that samples with high expression of ZNF503-AS2 were enriched in the MES subtype and CL subtype in the CGGA325 and CGGA693 databases. (TIF) [file pone.0314618.s002.tif]

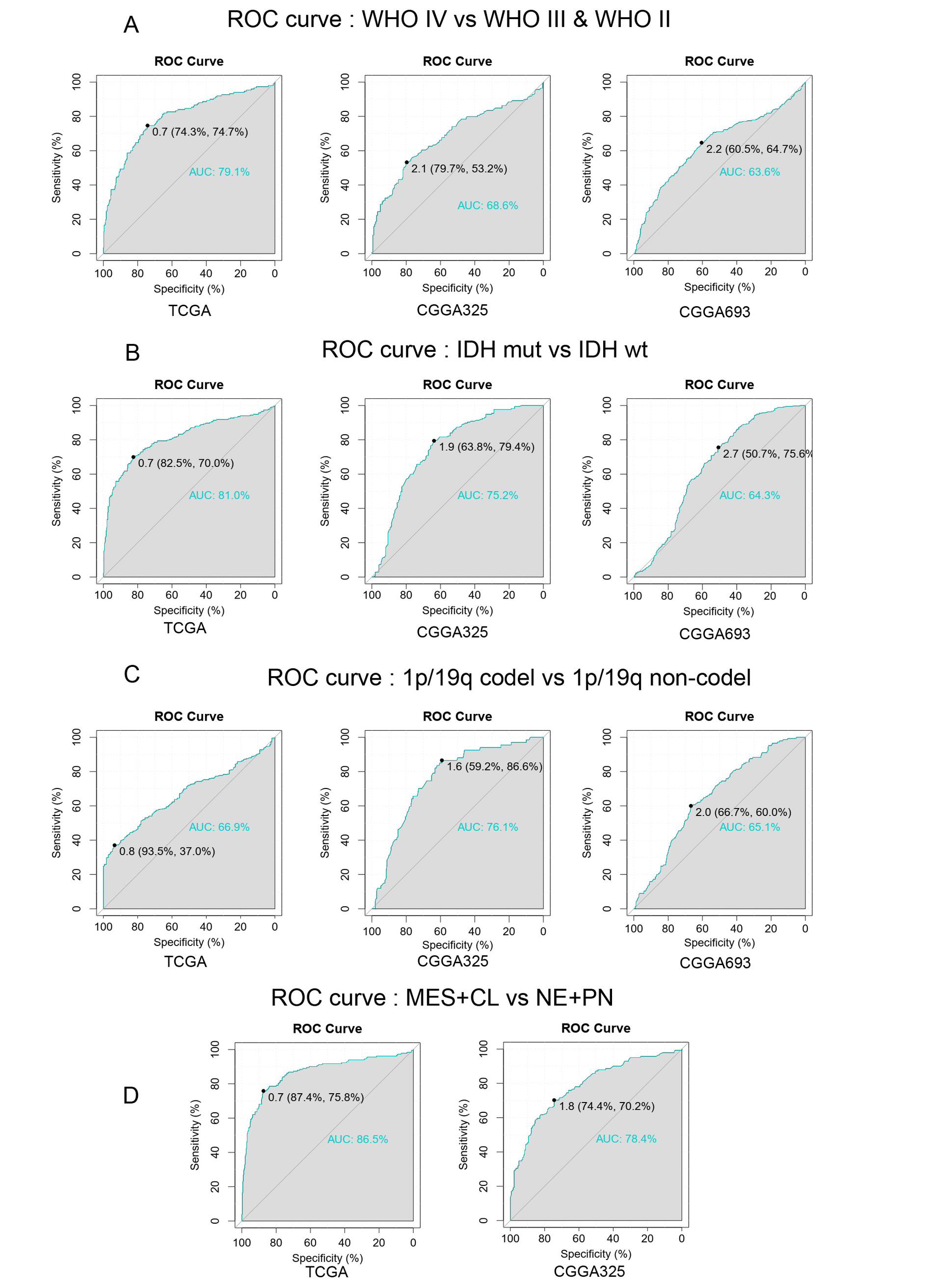

Supplement: S3 Fig — (A) ROC curve to assess the value of ZNF503-AS2 for GBM prediction. (B) ROC curve to assess the value of ZNF503-AS2 for IDH status prediction. (C) ROC curve to assess the value of ZNF503-AS2 for 1p/19q codeletion prediction. (D) ROC curve to assess the value of ZNF503-AS2 expression in predicting the MES molecular subtype and CL molecular subtype. (TIF) [file pone.0314618.s003.tif]

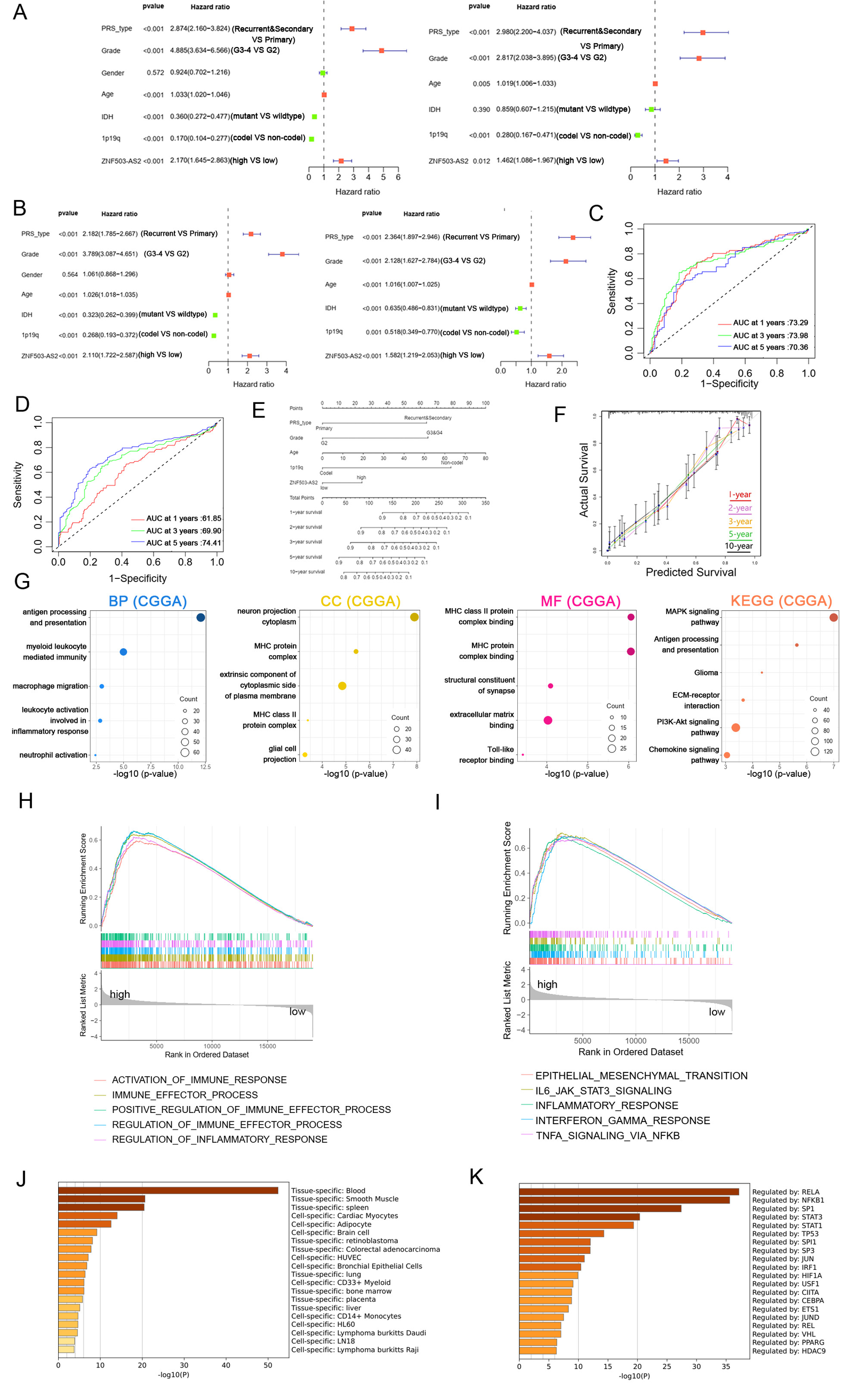

Supplement: S4 Fig — (A, B) Univariate and multivariate COX regression analysis of clinical data and ZNF503-AS2 expression in glioma samples in the CGGA325 database (A) and CGGA693 database (B). (C, D) Time-dependent ROC curves showed high predictive accuracy of ZNF503-AS2 for OS at 1, 3, and 5 years in glioma patients in the TCGA (C) and CGGA325 databases (D). (E) Nomogram combining ZNF503-AS2 and clinical characteristics predicts patient OS. (F) Calibration plots of predicted OS and actual OS. (G) Enrichment analysis of DEGs in the CGGA325 databases. (H, I) GSEA of DEGs in GO biological processes (H) and hallmark gene sets (I) in the CGGA325 databases. (J) PaGenBase shows that the DEGs are highly specifically expressed in the blood and spleen in the CGGA325 databases. (K) TRRUST shows that DEGs are mainly regulated by SP1, NFKB1 and RELA in the CGGA325 databases. (TIF) [file pone.0314618.s004.tif]

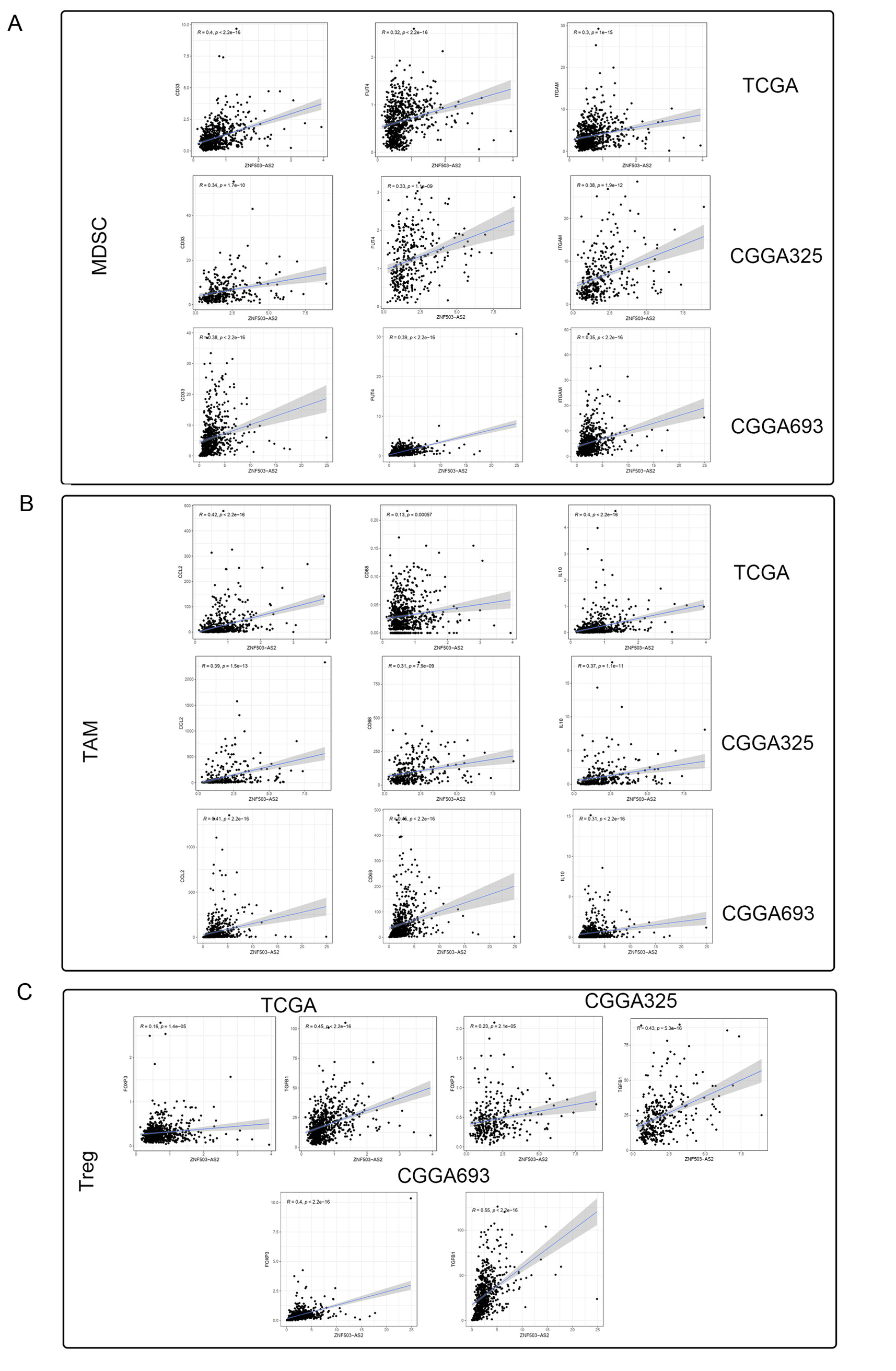

Supplement: S5 Fig — (A) Correlation of ZNF503-AS2 with markers of MDSCs. (B) Correlation of ZNF503-AS2 with markers of TAMs. (C) Correlation of ZNF503-AS2 with markers of Tregs. (TIF) [file pone.0314618.s005.tif]

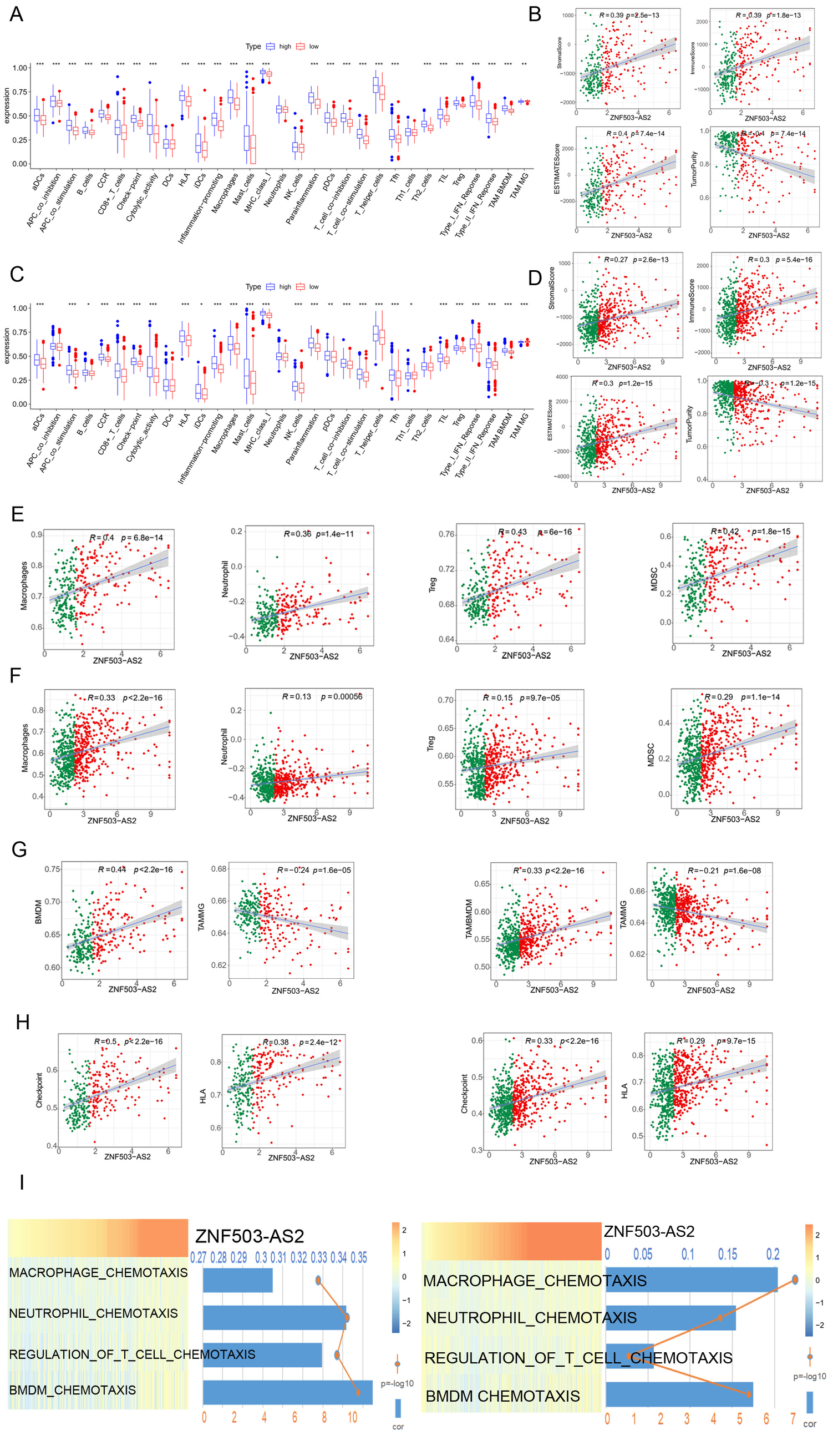

Supplement: S6 Fig — (A, C) ssGSEA showed significant differences in immune cell and immune function enrichment scores between the two groups in the CGGA325 (A) and CGGA693 databases (C). (B, D) Correlation analysis of stromal score, immune score, ESTIMATE score, and tumor purity with ZNF503-AS2 in the CGGA325 (B) and CGGA693 databases (D). (E, F) Correlation analysis of immunosuppressive cells with ZNF503-AS2 in the CGGA325 (E) and CGGA693 databases (F). (G) Correlation analysis of two TAMs with ZNF503-AS2 in the CGGA325 and CGGA693 databases. (H) ZNF503-AS2 is significantly and positively correlated with immune checkpoint and HLA enrichment scores in the CGGA325 and CGGA693 databases. (I) Heatmap of the correlation between chemotaxis of immunosuppressed cells and ZNF503-AS2 expression in the CGGA325 and CGGA693 databases. (TIF) [file pone.0314618.s006.tif]

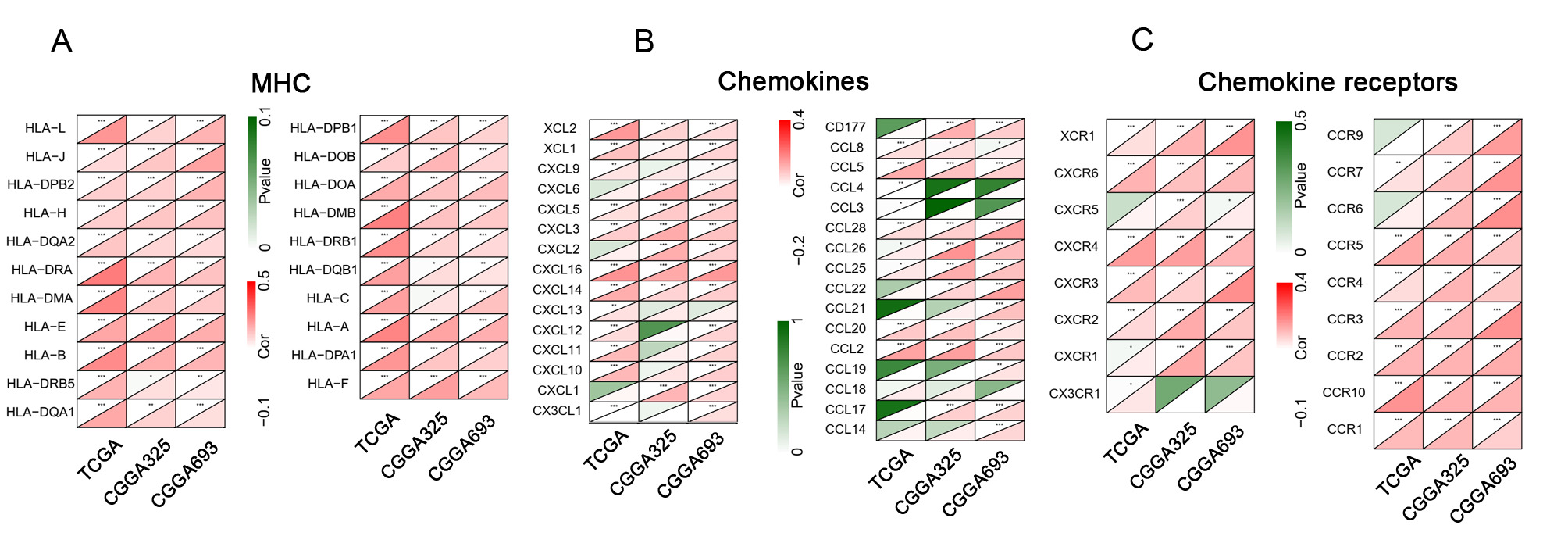

Supplement: S7 Fig — Correlation analysis of MHC molecules (A), chemokines (B), chemokine receptors (C) and ZNF503-AS2 expression. NS, not statistically significant; * P < 0.05; ** P < 0.01; *** P < 0.001. (TIF) [file pone.0314618.s007.tif]

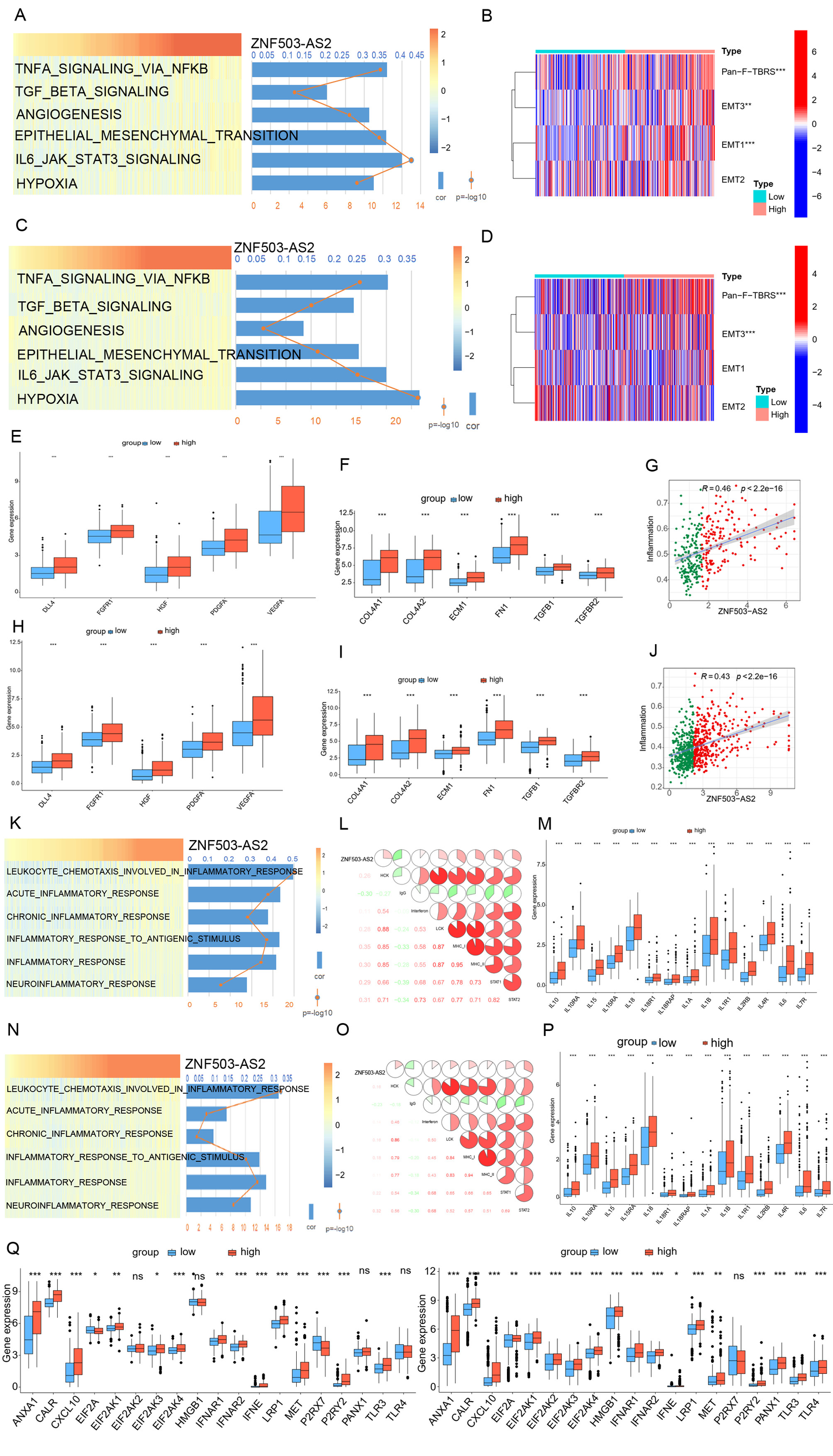

Supplement: S8 Fig — (A, C) Correlation between ZNF503-AS2 and the stromal activation pathway in the CGGA325 (A) and CGGA693 databases (C). (B, D) Multiple pathways were activated in the ZNF503-AS2 high-expression group in the CGGA325 (B) and CGGA693 databases (D). (E, H) Differential expression of angiogenic markers and pro-angiogenic factors between the two groups in the CGGA325 (E) and CGGA693 databases (H). (F, I) Differential expression of key molecules affecting lymphocyte infiltration between the two groups in the CGGA325 (F) and CGGA693 databases (I). (G, J) Enriched scores of inflammatory responses were significantly positively correlated with ZNF503-AS2 in the CGGA325 (G) and CGGA693 databases (J). (K, N) Correlation analysis of multiple inflammatory processes with ZNF503-AS2 in the CGGA325 (K) and CGGA693 databases (N). (L, O) Correlation analysis of multiple inflammatory processes with ZNF503-AS2 in the CGGA325 (L) and CGGA693 databases (O). (M, P) Multiple inflammatory factors were significantly different between the two groups in the CGGA325 (M) and CGGA693 databases (P). (Q) Differential expression of ICD-related genes. (TIF) [file pone.0314618.s008.tif]

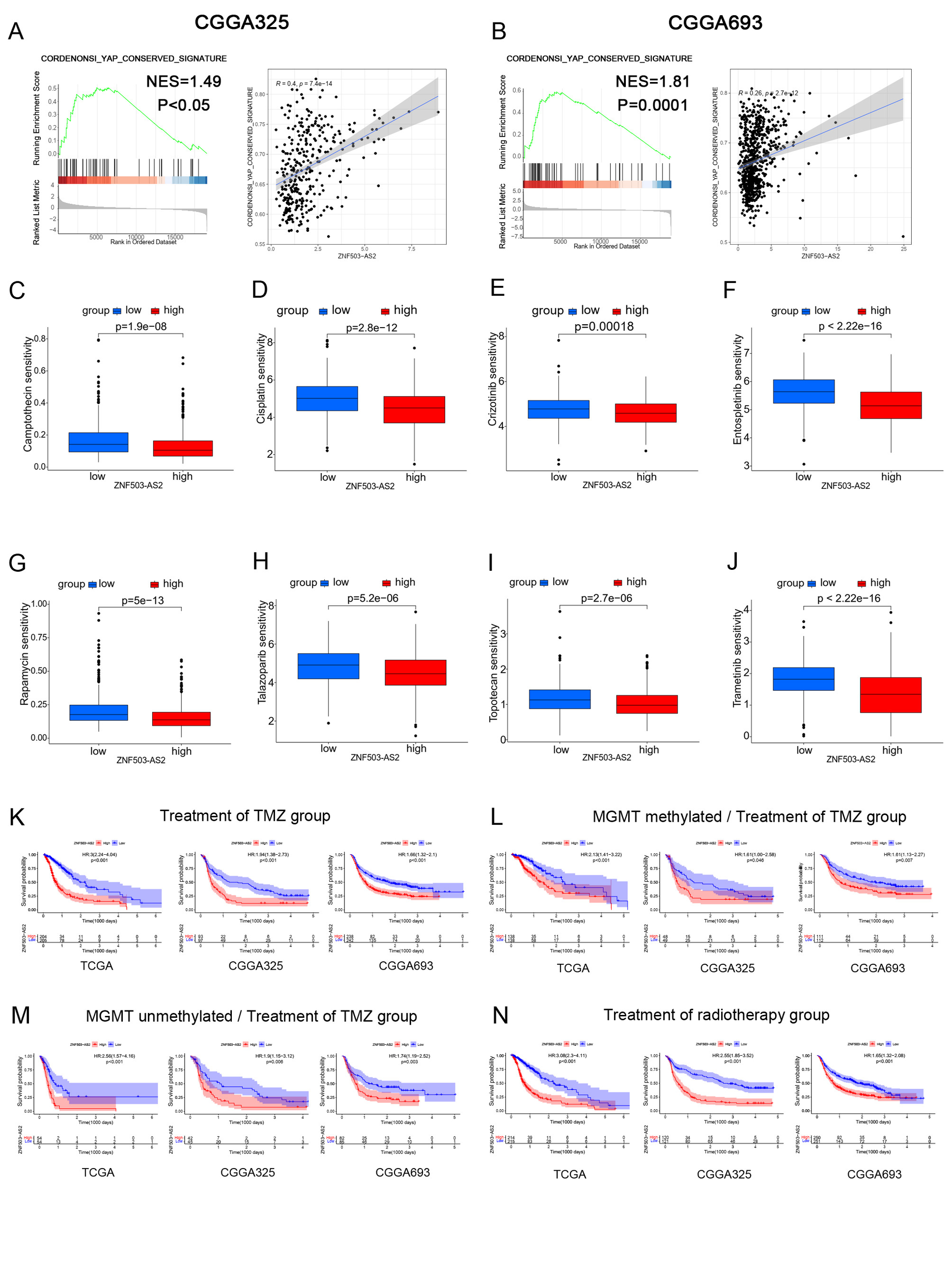

Supplement: S9 Fig — (A, B) ZNF503-AS2 high-expression samples were enriched in the YAP1 signaling pathway and positively correlated with the enrichment score of the YAP1 signaling pathway. (C-J) Multiple drugs are sensitive to patients with high ZNF503-AS2 expression. (K) Survival analysis of patients treated with temozolomide. (L, M) Survival analysis of temozolomide-treated patients according to whether the MGMT promoter is methylated (L) or not (M). (N) Survival analysis of patients receiving radiotherapy. (TIF) [file pone.0314618.s009.tif]

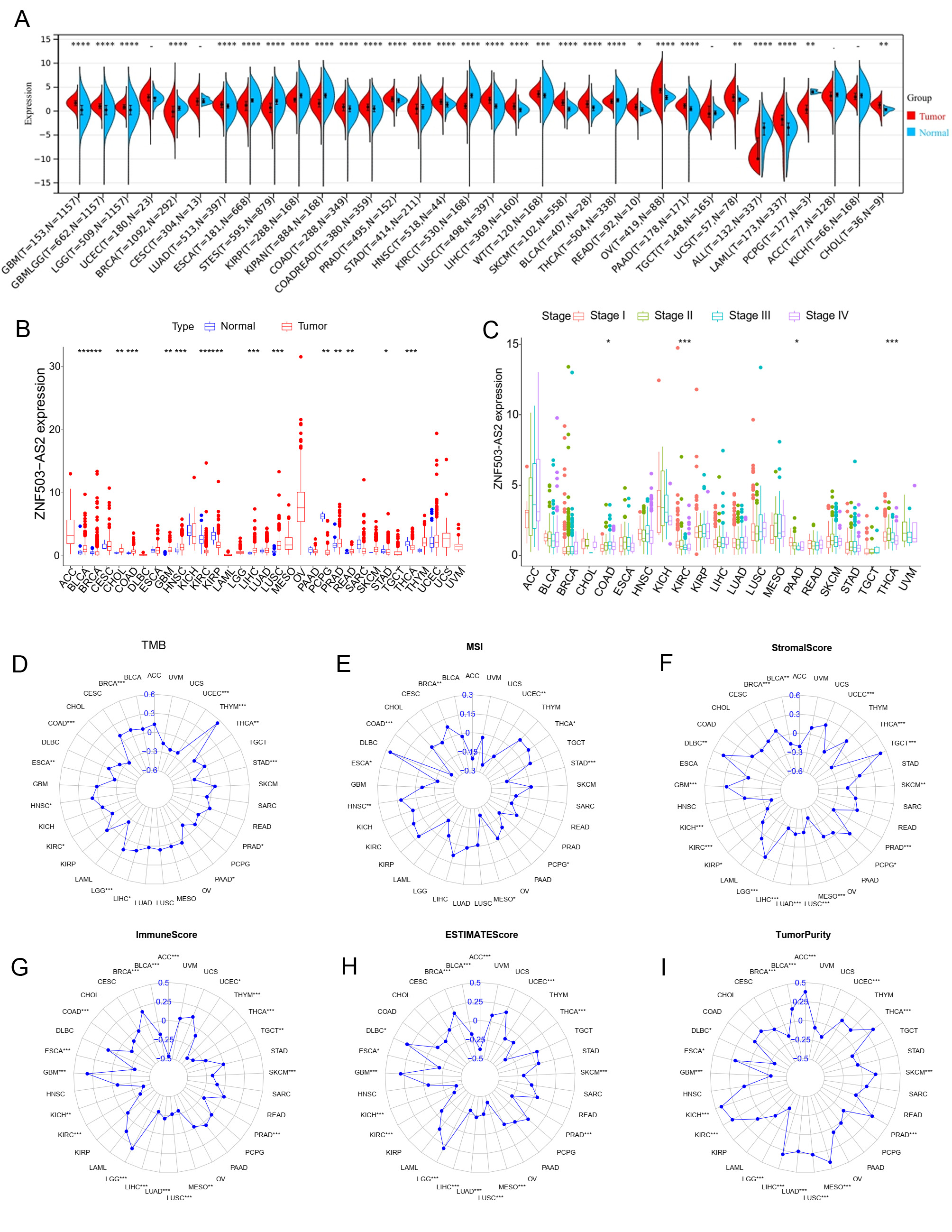

Supplement: S10 Fig — (A) ZNF503-AS2 expression in TCGA combined with GTEx. (B) ZNF503-AS2 expression in TCGA. (C) Relationship between ZNF503-AS2 expression and stage. (D-I) Correlation analysis between ZNF503-AS2 and TMB (D), MSI (E), stromal score (F), immune score (G), ESTIMATE score (H), and tumor purity (I). (TIF) [file pone.0314618.s010.tif]

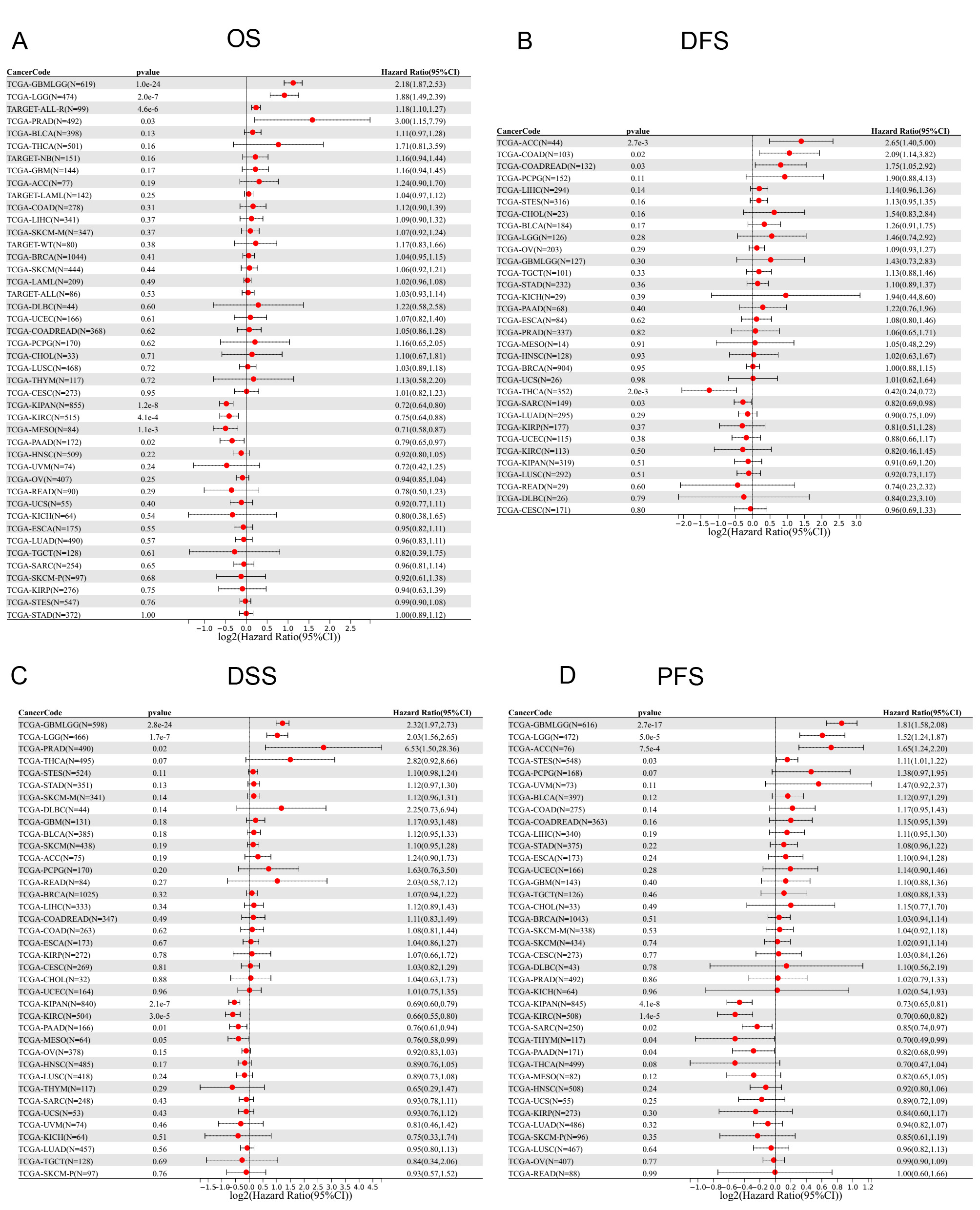

Supplement: S11 Fig — Univariate Cox regression analyses of OS (A), DFS (B), DSS (C) and PFS (D). (TIF) [file pone.0314618.s011.tif]

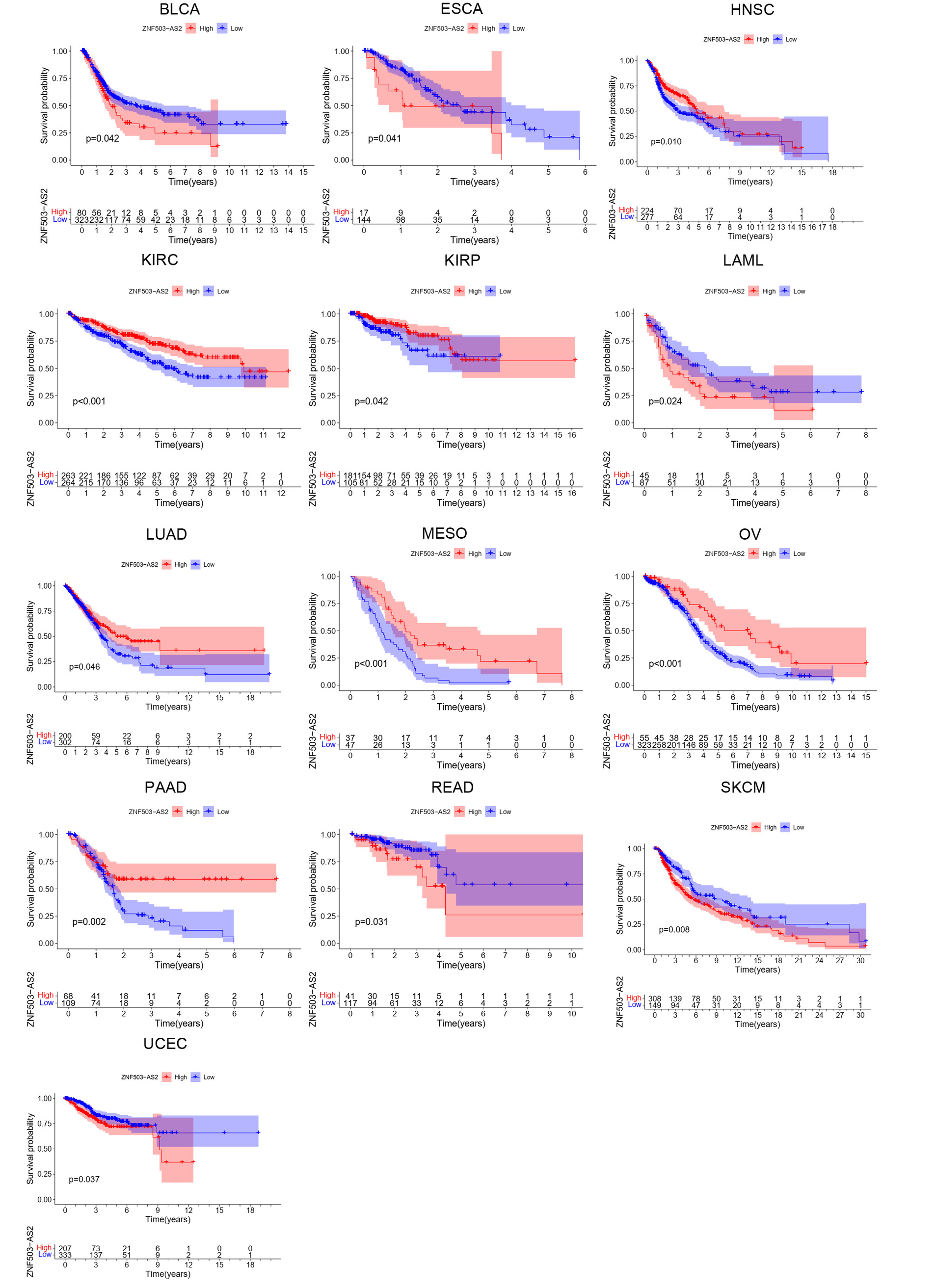

Supplement: S12 Fig — (TIF) [file pone.0314618.s012.tif]

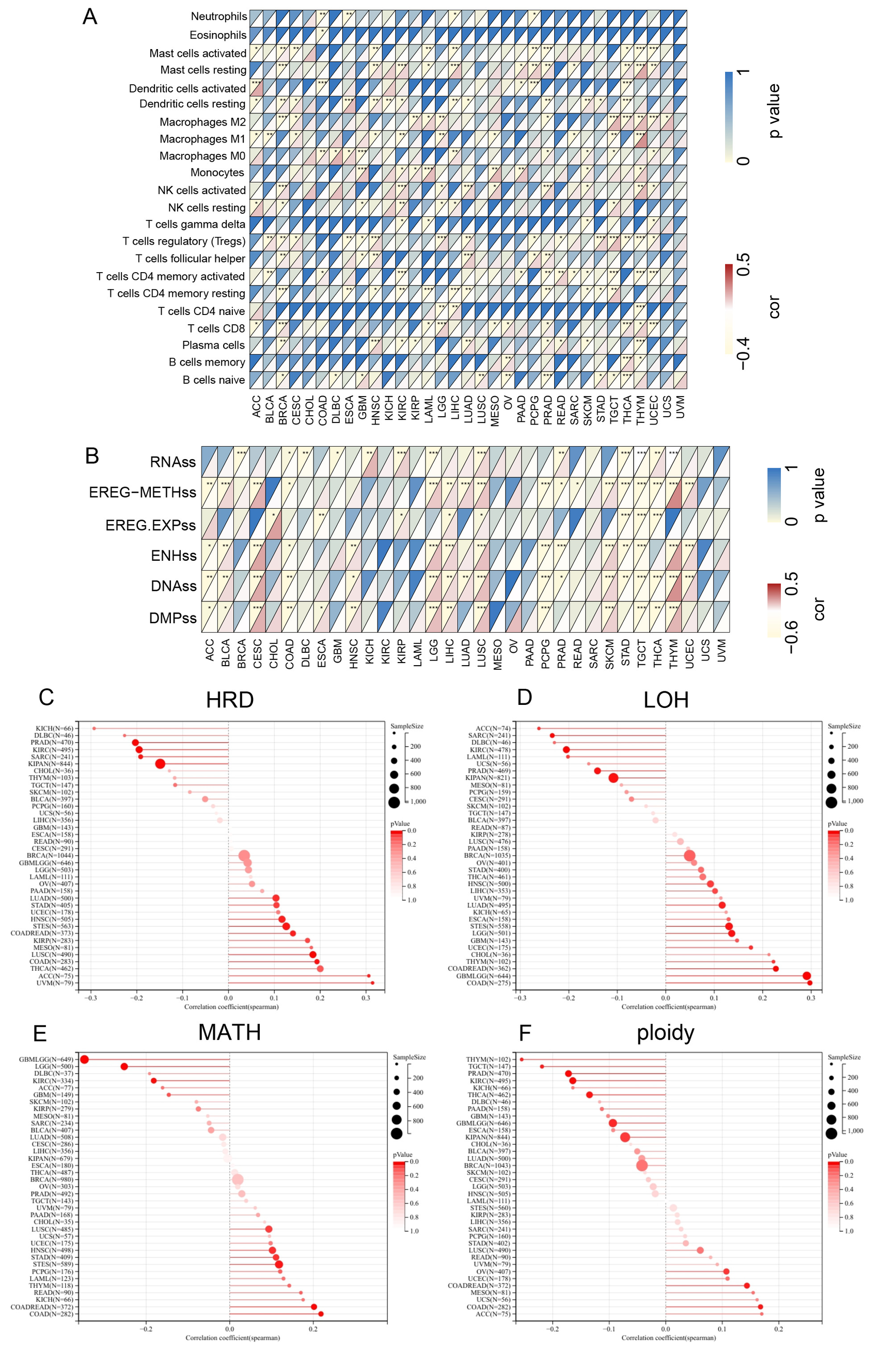

Supplement: S13 Fig — Correlation of ZNF503-AS2 with immune cell infiltration (A), cancer stemness index (B), HRD (C), LOH (D), MATH (E) and ploidy (F). (TIF) [file pone.0314618.s013.tif]
